# TFAP2E is implicated in central nervous system, orofacial and maxillofacial anomalies

**DOI:** 10.1136/jmg-2023-109799

**Published:** 2024-12-23

**Authors:** Jeshurun C Kalanithy, Enrico Mingardo, Jil D Stegmann, Ramgopal Dhakar, Tikam Chand Dakal, Jill A Rosenfeld, Wen-Hann Tan, Stephanie A Coury, Audrey C Woerner, Jessica Sebastian, Paul A Levy, Leah R Fleming, Lea Waffenschmidt, Tobias T Lindenberg, Öznur Yilmaz, Khadija Channab, Bimaljeet K Babra, Andrea Christ, Britta Eiberger, Selina Hölzel, Clara Vidic, Felix Häberlein, Nina Ishorst, Juan E Rodriguez-Gatica, Behnaz Pezeshkpoor, Patrick A Kupczyk, Olivier M Vanakker, Sara Loddo, Antonio Novelli, Maria L Dentici, Albert Becker, Holger Thiele, Jennifer E Posey, James R Lupski, Alina C Hilger, Heiko M Reutter, Waltraut M Merz, Gabriel C Dworschak, Benjamin Odermatt

**Affiliations:** 1 Institute of Neuroanatomy, Medical Faculty, University of Bonn, Bonn, Germany; 2 Institute of Human Genetics, University of Bonn, School of Medicine and University Hospital Bonn, Bonn, Germany; 3 Institute of Anatomy and Cell Biology, Medical Faculty, University of Bonn, Bonn, Germany; 4 Genome and Computational Biology Lab, Department of Biotechnology, Mohanlal Sukhadia University, Udaipur, Rajasthan, India; 5 Baylor Genetics Laboratories, Houston, Texas, USA; 6 Department of Molecular and Human Genetics, Baylor College of Medicine, Houston, Texas, USA; 7 Division of Genetics and Genomics, Boston Children’s Hospital, Boston, Massachusetts, USA; 8 Genomes2People Research Program, Division of Genetics, Department of Medicine, Mass General Brigham Inc, Boston, Massachusetts, USA; 9 Department of Pediatrics, Division of Genetic and Genomic Medicine, UPMC Children's Hospital of Pittsburgh, Pittsburgh, Pennsylvania, USA; 10 Department of Pediatrics, Children's Hospital at Montefiore, New York, New York, USA; 11 Genetics and Metabolic Clinic, St Luke's Health System, Boise, Idaho, USA; 12 Institute of Pharmaceutical Biology, Molecular, Cellular, and Pharmacobiology Section, University of Bonn, Bonn, Germany; 13 Clausius Institute of Physical and Theoretical Chemistry, University of Bonn, Bonn, Germany; 14 Institute for Experimental Hematology and Transfusion Medicine, University Hospital Bonn, Bonn, Germany; 15 Center for Rare Diseases Bonn, University Hospital Bonn, Bonn, Germany; 16 Department of Diagnostic and Interventional Radiology, University Hospital Bonn, Bonn, Germany; 17 Center for Medical Genetics, Ghent University Hospital, Ghent, Belgium; 18 Translational Cytogenomics Research Unit, Bambino Gesù Children's Hospital, IRCCS, Rome, Italy; 19 Medical Genetics Unit, Academic Department of Pediatrics, Bambino Gesù Children's Hospital, IRCCS, Rome, Italy; 20 Institute for Cellular Neurosciences II, University Hospital Bonn, Bonn, Germany; 21 Cologne Center for Genomics, University of Cologne, Cologne, Germany; 22 Molecular and Human Genetics; Human Genome Sequencing Center, Baylor College of Medicine, Houston, Texas, USA; 23 Texas Children’s Hospital, Houston, Texas, USA; 24 Research Center On Rare Kidney Diseases (RECORD), Erlangen University Hospital, Erlangen, Germany; 25 Department of Pediatrics and Adolescent Medicine, Friedrich-Alexander University Erlangen-Nuremberg, Erlangen, Germany; 26 Department of Pediatric and Adolescent Medicine, Division Neonatology and Pediatric Intensive Care, Friedrich-Alexander-Universität Erlangen-Nürnberg, Erlangen, Germany; 27 Institute of Human Genetics, Friedrich-Alexander University Erlangen-Nuremberg, Erlangen, Germany; 28 Department of Obstetrics and Prenatal Medicine, University Hospital Bonn, Bonn, Germany; 29 Department of Neuropediatrics, University Hospital Bonn, Bonn, Germany

**Keywords:** Hydrocephalus, Central Nervous System Diseases, Genetic Diseases, Inborn, Whole Exome Sequencing

## Abstract

**ABSTRACT:**

**Background:**

Previous studies in mouse, *Xenopus* and zebrafish embryos show strong *tfap2e* expression in progenitor cells of neuronal and neural crest tissues suggesting its involvement in neural crest specification. However, the role of human transcription factor activator protein 2 (*TFAP2E)* in human embryonic central nervous system (CNS), orofacial and maxillofacial development is unknown.

**Methods:**

Through a collaborative work, exome survey was performed in families with congenital CNS, orofacial and maxillofacial anomalies. Exome variant prioritisation prompted *TFAP2E* gene for functional analysis in zebrafish embryos. Embryonic morphology and development were assessed after antisense morpholino (MO) knockdown (KD), CRISPR/Cas9 knockout and overexpression of *tfap2e* in fluorescent zebrafish reporter lines using in vivo microscopy. Computational structural protein modelling of the identified human variants was performed.

**Results:**

In total, exome survey identified novel or ultra-rare heterozygous missense variants in *TFAP2E* in seven individuals from five independent families with predominantly CNS, orofacial and maxillofacial anomalies. One variant was found de novo and another variant segregated in an affected multiplex family. Protein modelling of the identified variants indicated potential distortion of TFAP2E in the transactivation or dimerisation domain. MO KD and CRISPR/Cas9 knockout of *tfap2e* in zebrafish revealed hydrocephalus and a significant reduction of brain volume, consistent with a microencephaly phenotype. Furthermore, mRNA overexpression of *TFAP2E* indicates dosage-sensitive phenotype expression. In addition, zebrafish showed orofacial and maxillofacial anomalies following *tfap2e* KD, recapitulating the human phenotype.

**Conclusion:**

Our human genetic data and analysis of Tfap2e manipulation in zebrafish indicate a potential role of *TFAP2E* in human CNS, orofacial and maxillofacial anomalies.

**WHAT IS ALREADY KNOWN ON THIS TOPIC**

- *Transcription factor activator protein 2 (TFAP2E)* has been delineated as a neural crest specifier and its expression in neural crest-derived tissues in zebrafish and *Xenopus* is well studied.

**WHAT THIS STUDY ADDS**

- Here, we identified five families with novel or ultra-rare heterozygous *TFAP2E* missense variants presenting with central nervous system (CNS), orofacial and maxillofacial anomalies.
- These phenotypes were recapitulated in our zebrafish knockdown and knockout models.
- Based on our functional and genetic data we propose *TFAP2E* to be implicated in the aetiology of human CNS, orofacial and maxillofacial anomalies.

**HOW THIS STUDY MIGHT AFFECT RESEARCH, PRACTICE OR POLICY**

- The present study provides human phenotype data for clinical genetics and characterises a novel syndromic disorder.
- Furthermore, our functional and protein modelling data may provide directions for future research on *TFAP2E*.

## Introduction

 The transcription factor activator protein 2 (TFAP2) family consists of five genes *TFAP2 A–E* that are closely linked to the regulation of neural crest cells (NCCs).^1^,^5^ Transcriptional regulation is carried out by homodimerisation or heterodimerisation of a TFAP2 protein member with its paralogs or various other transcription factors.^5^,^7^ Interestingly, the TFAP2 paralogs are conserved on protein level, with the highest sequence similarity in the C-terminal region (70%–80%).^8^

*TFAP2E* is known to interact in the downstream gene regulatory network of NCC specifiers that are induced by the neural plate border specifiers *PAX3* and *ZIC1*.^9^ As shown for TFAP2A/C and TFAP2A/B, heterodimerisation of TFAP2 paralogs facilitate chromatin access of neural plate border specifiers *PAX3, GATA1/3, ZIC1* as well as neural crest specifiers *SOX10, SOXD* and *FOXD3*.^5^,^7^ Additionally, TFAP2-activated genes show an enrichment of *SOXE*-binding and *MITF-*binding sites.^10^ Moreover, *TFAP2E* has been shown to perturb *DKK4* expression and consequently perturb WNT/ß-catenin signalling.^6^ In *Xenopus*, *tfap2e* was identified as a regulator of NCC specifiers *sox10* and *snail2* at the neurulation stage, and knockdown (KD) of Tfap2e in *Xenopus* resulted in expansion of the neural plate and loss of migratory NCCs.^9 11 12^

Previous expression studies in mice and *Xenopus* detected *Tfap2e/tfap2e* in the midbrain, hindbrain and rostral region of the spinal cord,^8 11 12^ in cranial NCCs migrating to the pharyngeal arches as well as in neural crest progenitor cells and Rohon-Beard sensory neurons (RBSNs).^13^ In zebrafish larvae (zfl), *tfap2e* expression was detected in the central nervous system (CNS) and NCCs including the presumed olfactory placode, medial telencephalon, hindbrain and melanocytes from 1 day postfertilisation (dpf) onwards.^14^

Dysregulation of neural plate border specification, as well as subsequent neural crest specification and migration, has been demonstrated to contribute to various CNS defects, neural tube defects^15^ and neurocristopathies, such as PCWH syndrome (MIM: 609136).^16^ However, up until now, *TFAP2E* has not been linked to any human congenital anomalies.

In the present study, we identified five unrelated families with novel or ultra-rare heterozygous variants in *TFAP2E* presenting with CNS, orofacial and maxillofacial anomalies. Depletion of Tfap2e in zfl resembled the human phenotypical spectrum in early zf morphant stages.

## Methods

### Human subjects and exome sequencing

Individual D-II:1 was part of an exome sequencing (ES) cohort of 208 unsolved cases with structural brain anomalies who underwent termination of pregnancy. Trio-ES was performed in family D as described previously.^17^ Candidate variants were filtered for novel or ultra-rare variants (Allele count: 1 or minor allele frequency (MAF) <0.0001) in Genome Aggregation Database gnomAD V.2.1.1 and an inhouse exome database.^17^ Following the pregnancy with D-II:4, ES of this individual was performed.

Individuals A-II:1, B-III:1, C-II:1 and E-II:1 were part of a database of individuals with neurodevelopmental disorder (NDD) who underwent clinical ES.^18^ In these individuals no pathogenic variant in an established disease gene was found on clinical evaluation and families were subjected to scientific evaluation. Candidate variants were filtered for novel or ultra-rare variants (MAF<0.0001) in gnomAD V.2.1.1. From all identified candidate variants, the *TFAP2E* variants were considered as potentially disease-causing and no other plausible candidate gene was shared between the affected individuals. For family B, segregation was performed by PCR and Sanger sequencing. For individuals A-II:1, C-II:1 and E-II:1 no parental DNA was available for segregation.

In order to look for copy number variations (CNVs) harbouring the *TFAP2E* locus, we reviewed entries in the Decipher database (https://decipher.sanger.ac.uk/), Baylor Genetics clinical chromosomal microarray database and conducted literature research for CNVs encompassing *TFAP2E* in 1p34.3 (online supplemental data 3 and 4 and online supplemental table 2).

### Computational 3D modelling of protein structure

Human TFAP2E variant mapping, 3D structure prediction and structural comparison were performed using the RaptorX web server (http://raptorx.uchicago.edu/ContactMap/) and Chimaera software V. 1.14 (onlinesupplemental data 5  6).^19^ We further used the ConSurf server to analyse conservation based on sequence analysis of *TFAP2E* homologues using default parameters.^20^

### *In silico* prediction of missense variants in *TFAP2E*

We used publicly available variant annotation tools for functional prediction of missense variant alleles, including Combined Annotation Dependent Depletion (CADD) GRCh38-v1.6, Polymorphism-Phenotyping V.2, Sorting Intolerant From Tolerant, EnsembleRegulatoryFeature^21^,^24^ and MetaDome V.1.0.1^25^ on the transcript ENST00000373235.3.

### Zebrafish husbandry and embryo maintenance

Zebrafish were raised and maintained according to national law and recommendations by Westerfield.^26^ Zfl of wild type (wt) AB/TL strain, transgenic *Tg(−3.1ngn1:GFP) (ZDB-FISH-1 50 901–25608*) and *Tg(sox10:mRFP) (ZDB-FISH-1 50 901–4058*) were obtained by natural spawning and raised at 28°C on a 14-hour light and 10-hour dark cycle.

### *In vivo* morpholino oligonucleotide and mRNA microinjections

KD of Tfap2e (ENSDART00000190005.1) was performed using specific morpholino (MO) oligonucleotides (GeneTools). We used one translation blocking (TB) and one splice blocking (e3i3) MO that have been established previously in a model for melanophore migration.^13^ In one-cell to two-cell embryos, we injected 3 ng of TB MO (5′-GCTGGAGTAGGAGTGGACTAACATC-3′; ZDB-MRPHLNO-1 01 013–3), 5 ng of e3i3 MO (5′-CACATGCAGACTCTCACCTTTCTTG-3′; ZDB-MRPHLNO-1 01 013–2) or 5 ng of control (Ctrl) MO (5'-CCTCTTACCTCAGTTACAATTTATA-3') into the yolk of the embryos. A pCMV-Sport6.1-based vector for human *TFAP2E* (ENST00000373235.4) as well as a pCMV-Sport6.1-based vector with the zebrafish orthologue *tfap2e* (ENSDART00000190005.1) were used for mRNA transcription followed by polyA-Tailing. For mRNA rescue experiments, we co-injected human *TFAP2E* mRNA in different amounts (between 10 pg and 250 pg) or 150 pg of MO-resistant zebrafish *tfap2e* mRNA together with Ctrl MO, TB MO or e3i3 MO. For a detailed list of reagents see online supplemental data 8.

To confirm the efficacy of MO KD, we performed western blot using a zebrafish-specific antibody detecting the C-terminal end of Tfap2e between aminoacids (aa) 303 µg and 330. 50 µg of zfl protein lysat was used per sample. Band density quantification was performed using Fiji ImageJ, normalised to housekeeping control Gapdh and finally adjusted to average blot intensities as previously described.^27^

Efficacy of e3i3 MO KD in zfl, was confirmed by rt-PCR using previously described methods.^28^ We used a pair of intron-spanning primers (forward, 5′-CACCACGGCCTGGATGATATT-3′; reverse, 5′-AGGACTCCTCCAAGCAGCGA-3′) to amplify *tfap2e* exon 2–exon 4, confirming the precise exclusion of exon 3, which leads to a frameshift and subsequently a premature stop codon (online supplemental data 7).

### CRISPR/Cas9-mediated *tfap2e* knockout in zebrafish

F0 *tfap2e* knockout (KO) zfl were generated by injection of a mix of four sgRNAs binding to exon 4 of *tfap2e*. Here, an insertion of 13 base pairs together with a deletion of 35 base pairs was confirmed by PCR and Sanger sequencing. The mutation is predicted to lead to a premature stop codon at position aa 199. F0 *tfap2e*^+/-^ KO^CRISPR^ zfl were bred with healthy wt zfl in F1 and F2 generation, and then outcrossed with transgenic *Tg(−3.1ngn1:GFP*) fish (*ZDB-FISH-1 50 901–25608*). For phenotypical evaluation, these heterozygous *tfap2e*^+/-^ zfl were incrossed to generate *tfap2e*^-/-^ KO^CRISPR^ zfl, *tfap2e*^+/-^ zfl and wt littermate control zfl. Genotyping of single embryos was performed by PCR amplification of the mutated region in exon 4 and subsequent allele-specific double digestion (online supplemental data 14).

### Fluorescence *in vivo* imaging and CNS/NC phenotype evaluation

Zfl were treated against pigmentation with 0.003% phenylthiourea from 1 dpf onwards. Phenotype evaluation was performed from 2 dpf to 5 dpf daily using a Zeiss Stemi 508 brightfield microscope. For statistical analysis, we compared the relative amount of unaffected zfl at 3 dpf in relation to the cumulative number of unaffected, affected and dead zfl until 3 dpf. A comparable amount of zfl (n) were used in each group for every independent experiment (N), as indicated in the respective graphs.

For detailed evaluation of the CNS phenotype, we in vivo imaged the zebrafish brain and spinal cord in *Tg(−3.1ngn1:GFP*)fluorescent reporter fish. To investigate the microencephaly phenotype, z-stack images were acquired, and brain volume was measured in a 3D model. Hydrocephalus was investigated by measuring the cleft between the forebrain hemispheres and normalised to the brain size in either the 3D model or in fluorescent whole mount brain images of KO^CRISPR^ zfl. Furthermore, we analysed the brain ventricles following in vivo microinjections of 1.7 nl of 20 µM sulforhodamine 101 into the diencephalic ventricle of *Tg(−3.1ngn1:GFP*) zfl at 2 dpf.

For evaluation of the neural crest phenotype, we manually counted RBSN, dorsal root ganglia (DRG) neurons and outgrowing central and peripheral projecting axons of the spinal cord over a length of 500 µm. Alcian blue cartilage staining was performed with zfl at 3 dpf using previously described methods to visualise the cartilage of craniofacial structures.^29^ Additionally, we evaluated NCC distribution in *Tg(sox10:mRFP*) zf outcrossed with wt AB/TL strain by fluorescence intensity analysis using Fiji/ImageJ (online supplemental data 9 and 14).

### Statistics

Two-tailed Student’s t-test, one-way or two-way analysis of variance test with Tukey’s multiple comparison and SEM were used for the respective analysis of treated zfl and controls. Data were first examined for normality using D’Agostino-Pearson and Shapiro-Wilk normalisation tests. Analysis of survival was performed using Kaplan-Meier survival curves and Mantel-Cox test. For all analyses, GraphPad Prism V.9.0.0 was used.

## Results

### Five families with novel or ultra-rare missense variants in *TFAP2E* show combined CNS, orofacial and maxillofacial anomalies

Following the identification of our index case with a de novo *TFAP2E* variant, we identified four additional families through personal communications with novel or ultra-rare missense variant alleles in *TFAP2E*, one of which was a multiplex family. In total we identified seven individuals from five families with novel or ultra-rare missense variants in *TFAP2E*. Four individuals had hydrocephalus (4/7), four had other CNS anomalies (4/7), two NDDs (2/5), and six had orofacial and maxillofacial anomalies (6/7) (table 1). The latter encompassed retrognathia/micrognathia (3/7) or cleft lip and palate (4/7). Detailed clinical reports and genetic data can be found in the online supplemental data 1 and 2.

**Table 1:** Phenotype data of individuals with heterozygous missense variants in *TFAP2E*

	Phenotype	A-II:1	B-I:2	B-II:4	B-III:1	C-II:1	D-II:1	E-II:1
CNS	Hydrocephalus	+			+		+	+
	Chiari II malformation	+						
	Corpus callosum agenesis	+						
	MMC	+						
	Thalamic lesion					+		
	Microlissencephaly						+	
	Hypoplastic cerebellum						+	
	Hypoplastic basal ganglia						+	
	Thinned brain stem						+	
	Alobar holoprosencephaly							+
NDD	ID/DD	+				+	n/a	n/a
	Hypotonia	+					n/a	n/a
	Attention-deficit/hyperactivity disorder					+	n/a	n/a
Craniofacial anomalies								
OMA	Retrognathia/micrognathia	+			+		+	
	Cleft lip and palate		+	+	+			+
Ear	Low-set ears				+			+
Nose	Prominent nasal bridge				+			+
	Short nose				+			+
	Abnormal nares				+			
Eye anomalies	Hypertelorism							+
	Deep-set eyes				+			
	Downslanting palpebral fissures				+			
Neurocranium	Macrocephaly					+		+
	Craniotabes							+
Limbs	Hypoplastic fifth toenails	+						
	Persistent digit pads	+						
	Single transverse palmar crease				+			
	Overlapping toe				+			
Heart	Ventricular septal defect				+			
	Atrial septal defect							+

CNScentral nervous systemDD, developmental delay; ID, intellectual disability; MMC, meningomyelocele; n/a, not assessable; NDD, neurodevelopmental disorder; OMA, orofacial and maxillofacial anomalyTFAP2Etranscription factor activator protein 2

Hydrocephalus was observed in four individuals: Individuals A-II:1 and E-II:1 developed secondary hydrocephalus as a result of Chiari II malformation (A-II:1) or alobar holoprosencephaly (E-II:1). Individual B-III:1 from family B presented with communicating external hydrocephalus without signs of occlusion. Similarly, individual D-II:1 presented prenatal with communicating external hydrocephalus without signs of occlusion.

CNS anomalies were observed in four individuals: Individual A-II:1 presented with Chiari II malformation, agenesis of the corpus callosum and myelomeningocele. Individual C-II:1 presented with a thalamic lesion of uncertain origin. Individual D-II:1 presented with microlissencephaly, hypoplastic cerebellum, hypoplastic basal ganglia and thinned brain stem. Individual E-II:1 presented with alobar holoprosencephaly.

Orofacial and maxillofacial anomalies were observed in six individuals: Individuals A-II:1, B-III:1 and D-II:1 presented with retrognathia/micrognathia. Of note, three individuals of the multiplex family B presented with cleft lip and palate (B-I:2, B-II:4, B-III:1), as well as individual E-II:1.

NDDs were observed in two individuals: Individual A-II:1 presented with intellectual disability and hypotonia, individual C-II:1 presented with intellectual disability and attention deficit/hyperactivity disorder. NDD could not be assessed in two individuals since this was not possible in the prenatal situation nor in the deceased neonate.

**Figure 1:**
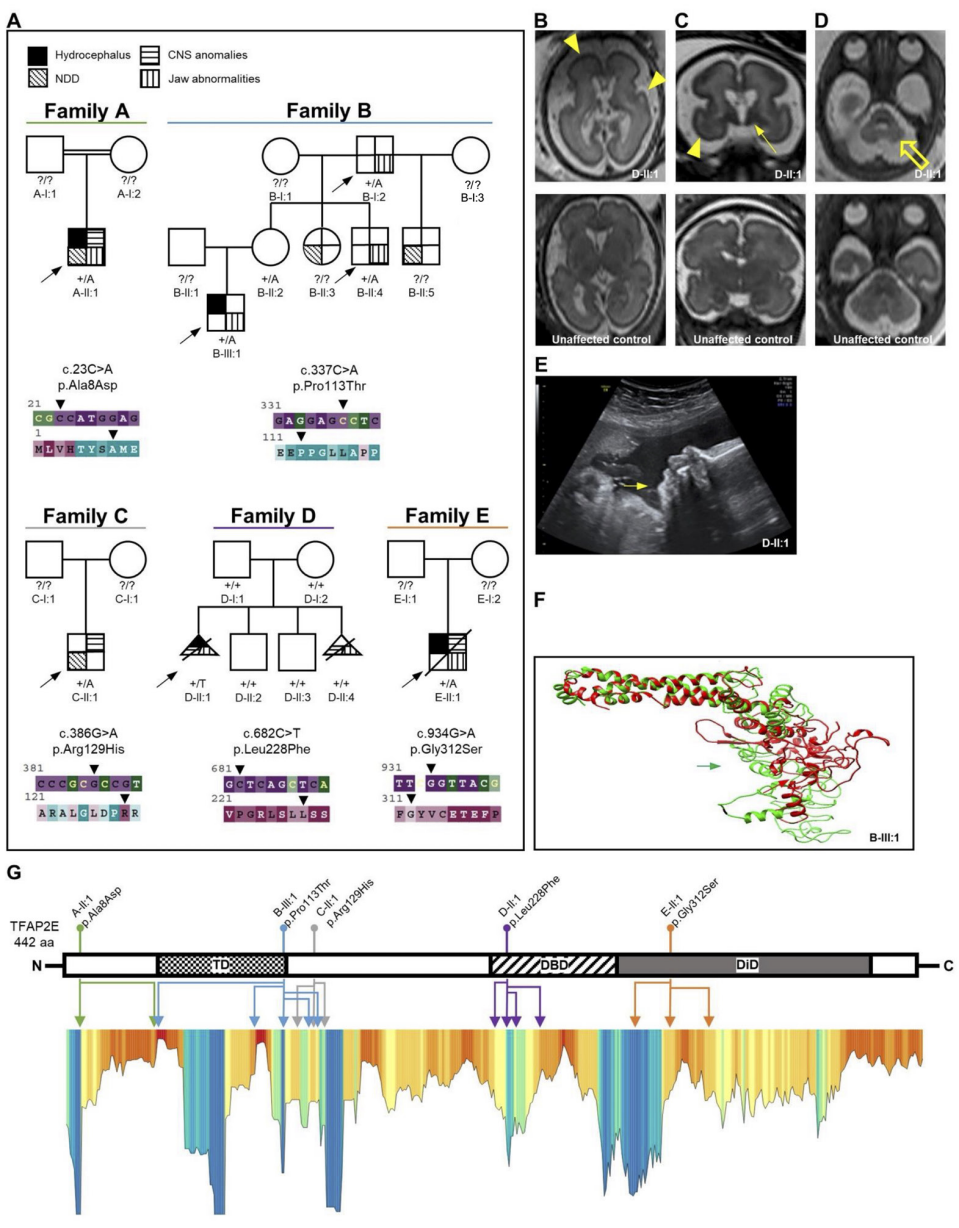
Clinical and molecular data of families with heterozygous transcription factor activator protein 2 (*TFAP2E)* missense variants. (**A**) Family pedigrees of individuals with heterozygous variants in *TFAP2E* and respective ConSurf conservation analysis for base pair (upper row) and amino acid (aa) conservation (lower row). Affected individuals are indicated with different shapes for each phenotype according to the legend. Arrowheads indicate the respective variant position. Purple/red colour indicates high conservation of the respective residue. Note the multiplex family B with overlapping orofacial and maxillofacial and neurodevelopmental phenotype (table 1, online supplemental data 1) and the de novo variant in the female individual D-II:1. The arrows indicate probands. Filled shapes reflect affected status. (**B–D**) Fetal brain MRI of D-II:1 (upper panel) compared with that of a healthy subject with similar gestational age as reference (lower panel). Besides the obvious microcephaly, note the reduced sulcation with smooth brain surface characteristic of lissencephaly (B, C, yellow arrowheads). Furthermore, an overall abnormal signal intensity of subcortical white matter as well as hypotrophy of basal ganglia is evident (C, yellow solid arrow). The suspected cerebellar hypoplasia was seen in the axial plane (D, yellow hollow arrow). (**B**) Axial T2 TSE (turbo spin echo), (**C**) coronal SSFP (steady-state free precession), (**D**) axial SSFP. (**E**) Ultrasound imaging of fetus D-II:1 at gestational week 30+4 shows severe retrognathia (white arrow). (**F**) Computational 3D protein modelling of TFAP2E showing the native prediction (green) and the variant prediction of B-III:1 (red). The variant site is marked by the respective (green/red) arrow. The missense variant changes the polarity of the aa residue, leading to conformation changes of the neighbouring aa. This completely changes the 3D structure of the affected transactivation domain. (**G**) Linearised visualisation of the TFAP2E protein and lollipop chart indicating the localisation of the individuals’ aa variants, which are colour-coded according to the family pedigrees in A. The identically colour-coded double-headed arrows below indicate the multiple positions of the variants’ structural effects predicted by our protein modelling data. Lower panel: Metadome Protein Tolerance Landscape indicating the residues with general intolerance to structural variations (orange–red colour indicates high intolerance). Note that for some variants a secondary effect to a neighbouring position and not the variant location itself are in a region of strong intolerance. That is true for p.Ala8Asp (A-II:1), p.Pro113Thr (B-III:1) and p.Leu228Phe (D-II:1) (online supplemental table 1). CNS, central nervous system; DBD, DNA-binding domain; DiD, dimerisation domain; NDD neurodevelopmental disorder; TD transactivation domain.

In the multiplex family B the novel *TFAP2E* variant c.337C>A was identified in individual B-III:1 using ES. The c.337C>A variant segregated in the affected family members B-I:2, B-II:4 and B-III:1 over three generations. However, the mother (B-II:1) did not show congenital CNS, orofacial and maxillofacial anomalies, but she had chronic migraine and an anxiety disorder. Moreover, a maternal aunt (B-II:3) and a maternal half-uncle (B-II:5) (related to the maternal grandfather) presented with intellectual disability, but their DNA was not available for segregation analysis (figure 1A, table 1, online supplemental data 1).

In family D, Trio-ES revealed a novel de novo *TFAP2E* variant (c.682C>T) (figure 1B–E, table 1). Notably, the parents of D-II:1 had in a later pregnancy a fetus (D-II:4) with cerebellar hypoplasia and retrognathia only (online supplemental data 2). On ES, the *TFAP2E* variant c.682C>T was not found in D-II:4. Pathogenic variants in known disease genes could be ruled out in the two fetuses (online supplemental data 1), they shared no other plausible candidate variant and we did not identify an independent genetic factor in individual D-II:4.

### Missense variants in *TFAP2E* disrupt neighbouring functional domains

In seven individuals from five families with a combined CNS/orofacial and maxillofacial phenotype, we identified novel or ultra-rare heterozygous *TFAP2E* missense variant alleles (figure 1A). Four out of five (4/5) variant alleles were predicted to be damaging with a CADD Score above 23 (table 2).

**Table 2:** Molecular data of *TFAP2E* variant alleles

Family	Family A	Family B	Family C	Family D	Family E
Zygosity	Heterozygous	Heterozygous	Heterozygous	Heterozygous	Heterozygous
Inheritance	n/a	Maternally inherited	n/a	De novo	n/a
Family history	Consanguinity	Multiple affected family members		One sibling with partly overlapping, milder phenotype, but no variant in *TFAP2E*	
Chr1 (NC_000003.12)	g.35573600	g.35574236	g.35574285	g.35588449	g.35590663
*TFAP2E* (NM_178548.4)	c.23C>A	c.337C>A	c.386G>A	c.682C>T	c.934G>A
TFAP2E (UniProt Q6VUC0)	p.Ala8Asp	p.Pro113Thr	p.Arg129His	p.Leu228Phe	p.Gly312Ser
Exon (ENST00000373235.4)	1/7	2/7	2/7	4/7	6/7
CADD PHRED (GRCh38-v1.6)	23.50	15.18	23.50	25.80	24.10
SIFT	Deleterious	Tolerated	Tolerated	Deleterious	Deleterious
PolyPhen-2	Possibly damaging (0.845)	Possibly damaging (0.601)	Probably damaging (0.979)	Probably damaging (0.993)	Possibly damaging (0.721)
EnsemblRegulatoryFeature	Promoter	Promoter	Promoter	n/a	n/a
GnomAD V.2.1.1, MAF	Novel	Novel	Novel	Novel	MAF 0.00003 in Latino/admixed American population; allele count: 1; number of homozygotes: 0
Conservation of aa residue in zebrafish (see also online supplemental data 10)	Not conserved	Not conserved	Conserved	Conserved	Conserved

aa, amino acid; CADDCombined Annotation Dependent DepletionID, intellectual disabilityMAF, minor allele frequency; n/a, not available; PolyPhen-2Polymorphism-Phenotyping V.2SIFTSorting Intolerant From TolerantTFAP2Etranscription factor activator protein 2

All investigated variants map to highly conserved residues at the base pair level (figure 1A), including three variants c.23C>A (family A), c.337C>A (family B) and c.386G>A (family C) residing in a predicted internal promoter region of the *TFAP2E* coding region, according to EnsembleRegulatoryFeature.^23^

The protein sequence of human TFAP2E (Q6VUC0) and mouse TFAP2E (Q6VUP9) show 93% similarity, allowing us to map the previously described murine functional domains to human TFAP2E (figure 1G upper panel, online supplemental data 10). Next, we combined our computational 3D protein modelling data with the MetaDome Tolerance Landscape, which evaluates the tolerance to structural variation of the respective aa residues. This approach revealed intolerance for modification of the secondary structure of the variant residues p.Arg129His (family C) and p.Gly312Ser (family E). Of note, even though the not conserved residues p.Ala8Asp (family A), p.Pro113Thr (family B) and the conserved residue p.Leu228Phe (family D) are rated as (highly) tolerant to structural variation, our 3D model predicts distortion of structurally neighbouring regions to be (highly) intolerant to changes (online supplemental table 1, figure 1G, onlinesupplemental data 5  6). For instance, the variant p.Pro113Thr (family B) leads to a change in polarity and might possibly alter structure and activity of the neighbouring transactivation domain (p.50, p.96–99) (figure 1F,G).

### Expression profile of *tfap2e* and establishment of zfl models with different *tfap2e* gene dosage

**Figure 2:**
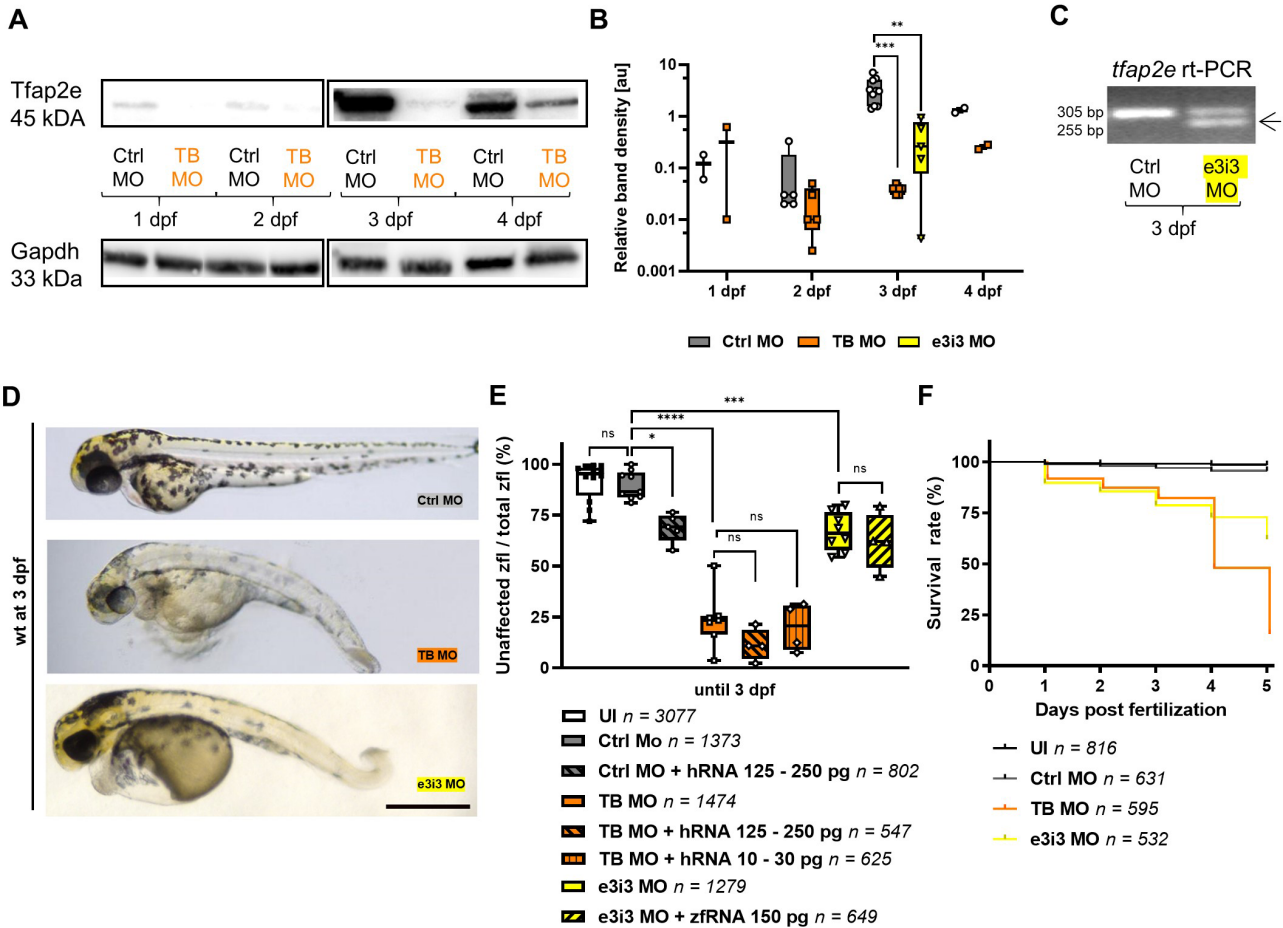
Tfap2e morpholino KD model in zfl. (**A–B**) Chemiluminescence bands and box plot graph of western blots of zfl on Tfap2e morpholino (MO) KD and controls from 1–4 days post fertilisation (dpf). Each dot on the box plot graph represents an independent experiment with 50 µg zfl protein of an independent injection. Band intensities were plotted on a logarithmic scale. Note the increasing genuine Tfap2e expression between 2 dpf and 3 dpf and the significantly suppressed expression on TB and e3i3 MO KD at 3 dpf. The housekeeping control Gapdh shows a consistent expression over all timepoints. The uncropped western blot membrane pictures are provided in online supplemental data 11. (**C**) Agarose gel electrophoresis after rt-PCR on *tfap2e* at 3 dpf. Tfap2e KD with e3i3 MO leads to an alternative, 50 bp shorter transcript (arrow) with precise exclusion of exon 3. This was further confirmed by Sanger sequencing (online supplemental data 7). (**D–E**) Representative brightfield microscopy images of zfl and box plot graph on the rate of unaffected zfl on Tfap2e KD. Each dot in the box plot graph indicates an independent injection experiment. Wild type (wt) strain zfl on *tfap2e* MO KD at 3 dpf present with microcephaly, scoliosis and hypopigmentation. The KD results in a significant reduction of phenotypically unaffected zfl. Co-injection of wt human RNA or zebrafish RNA (zfRNA) in morphants does not rescue the phenotype. Moreover, overexpression of *TFAP2E* by wt *TFAP2E* human polyA mRNA (hRNA) injection into Ctrl MO zfl leads to expression of the morphant phenotype in some of these controls. (**F**) Kaplan-Meier survival curve of *tfap2e* MO KD zfl. Both morphants show a poorer survival rate with a rapidly decreasing survival after 3 dpf especially for the TB MO KD, congruent with the timepoint of physiological Tfap2e upregulation. n=4; p<0.0001 (Mantel-Cox test). Ns, not significant; *p<0.05 **; p<0.01; ***p<0.001; ****p<0.0001 (B, E: one-way analysis of variance (ANOVA) with Tukey’s multiple comparison). Number of investigated zfl *n* as indicated. Scale bar: 500 µm. KD, knockdown; TB, translation blocking; TFAP2E, transcription factor activator protein 2; zfl, zebrafish larvae.

We initiated functional studies in zebrafish given an aa sequence similarity of 77% comparing human TFAP2E to zebrafish Tfap2e (Q6P0E7) (online supplemental data 10). Protein quantification of Tfap2e in whole zfl lysates showed a dynamic and accurate regulation of protein expression levels especially from 2 dpf to 3 dpf (figure 2A,B, online supplemental data 11). In a next step, we performed MO-based KD of Tfap2e.^14^ We were able to confirm KD efficacy of Tfap2e in morphants through western blot in which we observed nearly complete depletion of the Tfap2e protein at 3 dpf (figure 2A, upper panel, figure 1B). KD with the e3i3 MO was proven by rt-PCR (figure 2C, online supplemental data 7). In a parallel approach, a *tfap2e* KO^CRISPR^ zebrafish line was generated.

The most striking phenotypes in our MO-KD zfl at 3 dpf were microcephaly, scoliosis and hypopigmentation (the latter was previously described by van Otterloo *et al*)^14^ (figure 2D). Only 24% of TB MO and 67% of e3i3 MO morphants showed normal healthy phenotype, compared with 90% of controls. Overexpression of *TFAP2E* with human wt mRNA in Ctrl MO zfl partly replicated the morphant phenotype: only 69% of these zfl remained unaffected after 3 dpf, compared with 90% of zfl treated only with Ctrl MO (figure 2E). Consequently, co-injection of wt human or zf mRNA in different concentrations on Tfap2e KD could not significantly rescue the number or features of phenotypical expression.

Interestingly, the survival rate of morphant zfl rapidly reduced from 3 dpf onwards, the timepoint of upregulated Tfap2e expression. TB MO showed a lower survival rate (16 %) compared with e3i3 MO (62 %) at 5 dpf, congruent with the manifestation of a more severe phenotype (figure 2F).

### Depletion of Tfap2e in zfl leads to a syndromic phenotype with hydrocephalus, neuronal migration deficit, orofacial and maxillofacial anomalies, and microcephaly

**Figure 3:**
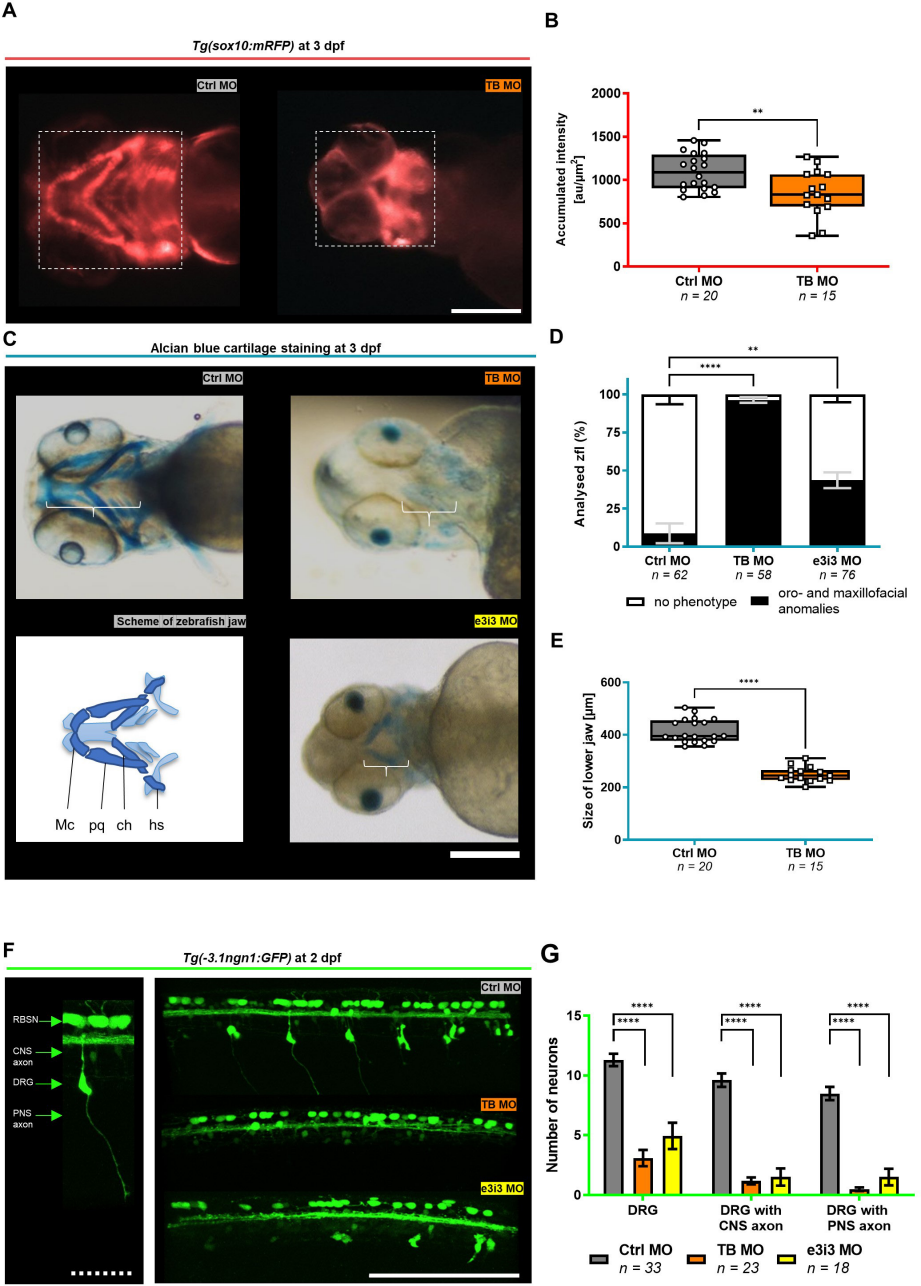
Tfap2e KD is implicated in the disruption of neural crest-specific tissues. (**A–B**) Representative in vivo fluorescence imaging of the jaw of *Tg(sox10:mRFP*) zfl at 3 days post fertilisation (dpf) on Tfap2e KD and box plot graph of sox10:mRFP reporter-signal intensity. The dotted rectangle indicates the area that was chosen to measure the accumulated fluorescence intensity in arbitrary units (au)/µm^2^. Morphants present with a pathological formation and a reduced Sox10 signal at 3 dpf after Tfap2e KD, indicating neural crest cell disruption. (**C–E**) Representative Alcian blue cartilage staining of zfl at 3 dpf and simplified schematic of the developing zebrafish craniofacies (**C**), column graph of the percentage of zfl with orofacial and maxillofacial anomalies (**D**), and box plot graph of measured sizes of the lower jaw on Tfap2e KD (**E**). White brackets in (**C**) indicate the measured distance from the rostral end of the Meckel’s cartilage to the caudal point of the hyosympletic cartilage. On Tfap2e KD, we observed a reduced jaw size according to hypoplasia of relevant orofacial and maxillofacial structures including the Meckel’s cartilage (Mc), ceratohyal (ch), palatoquadrate (pq) and hyosympletic (hs). (**F–G**) Representative in vivo 2-photon microscopy of the spinal cord of *Tg(−3.1ngn1:GFP*) zfl on Tfap2e KD at 2 dpf and column graph of counted fluorescent neurons and their axons. The left panel is a close-up view and description of the analysed cell types. Note the strongly reduced number of DRG neurons in both morpholino (MO) KD groups. Additionally, central and peripheral axonal outgrowth of the remaining DRG neurons is impaired. **p<0.01, ****p<0.0001 (B, E: unpaired *t*-test; D: one-way ANOVA with Tukey’s multiple comparison; G: two-way ANOVA with Tukey’s multiple comparison). Number of independent experiments n=3 for all graphs, number of investigated zfl *n* as indicated. White scale bars: 200 µm. White dotted scale bar: 50 µm. ANOVA, analysis of variance; CNS, central nervous system; DRG, dorsal root ganglia; KD, knockdown; TB, translation blocking; zfl, zebrafish larvae.

As NCCs are necessary for neuronal and also non-neuronal tissue formation, that is, orofacial and maxillofacial cartilage, we investigated the morphology of these structures in 3 dpf zfl in the fluorescent neural crest reporter line *Tg(sox10:mRFP*). Morphants presented with a pathological formation of anterior NCCs in the orofacial and maxillofacial region at 3 dpf. The mRFP signal intensity was reduced from 1096 au/µm² (Ctrl MO) to 851 au/µm² (TB MO), indicative of disrupted expression of *sox10* in the morphant neurocranium and viscerocranium (figure 3A,B). To further support these findings, we assessed Alcian blue cartilage staining of whole zfl. The Meckel’s cartilage, palatoquadrate, ceratohyal or hyosympletic bone showed severe defects after Tfap2e KD with almost complete penetrance (96 %) in TB MO and high penetrance (44 %) in e3i3 MO zfl. The size of the lower jaw was accordingly reduced from 415 µm (Ctrl MO) to 250 µm (TB MO) (figure 3C,D,E).

Assessment of trunk NCC migration was performed by in vivo imaging of DRG neurons and their precursor population RBSN in the spinal cord of *Tg(−3.1ngn1:GFP*) fluorescent reporter zfl at 2 dpf. The number of DRG neurons was drastically reduced in both groups (TB MO: 3.1, e3i3 MO: 4.9, Ctrl MO: 11.3 neurons). Remarkably, the remaining bipolar DRG neurons showed an impaired axonal outgrowth independent of the direction towards CNS or peripheral nervous system (PNS) (CNS - TB MO: 1.2; e3i3 MO 1.5; Ctrl MO: 9.6 axons / PNS - TB MO: 0.5; e3i3 MO 1.5; Ctrl MO: 8.5 axons) (figure 3F,G). However, manual counting of neuron somata did not reveal a significant alteration in RBSN population on MO KD (online supplemental data 12).

**Figure 4:**
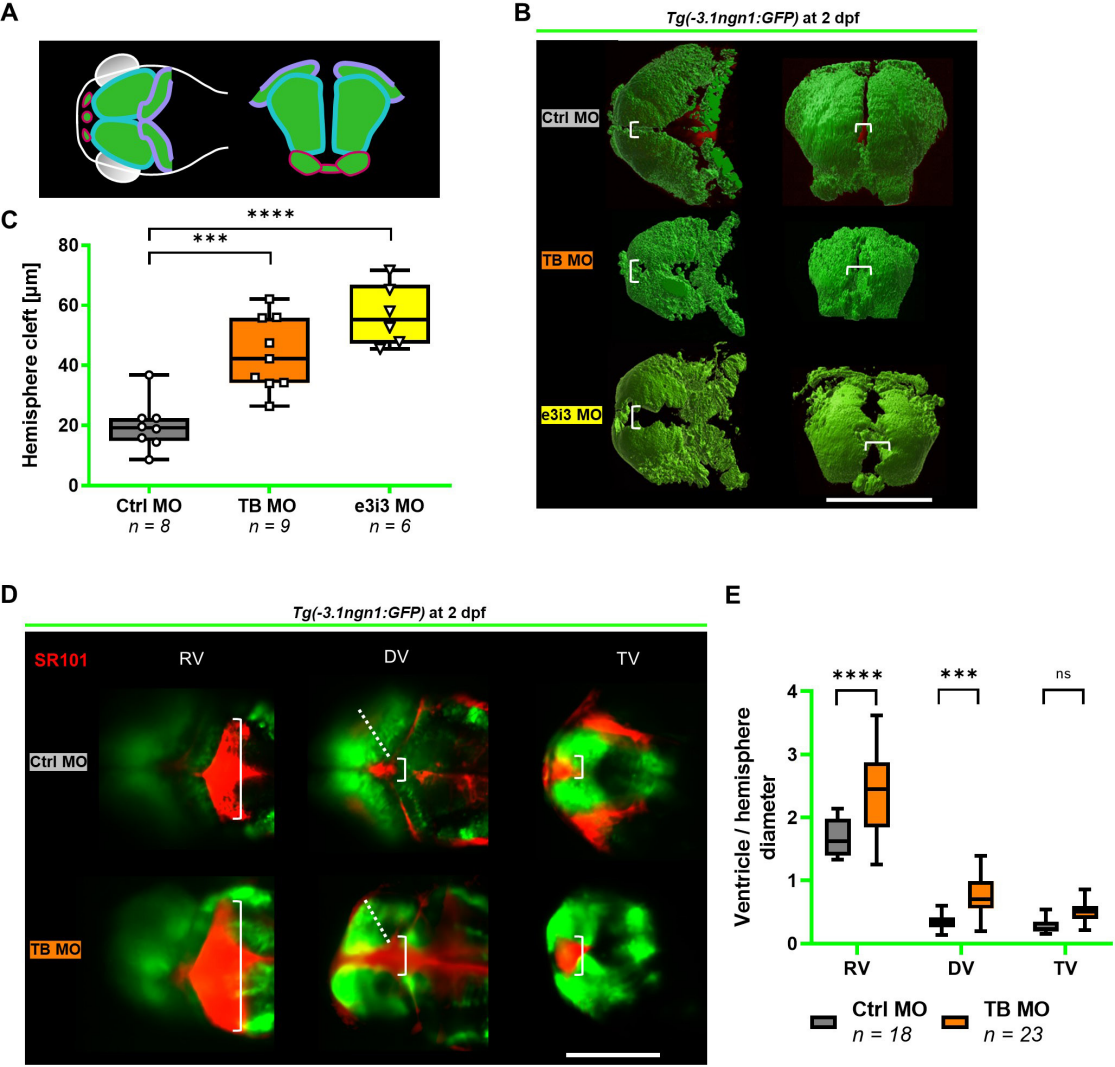
Tfap2e KD is implicated in structural brain anomalies in the early developing zfl. (**A–C**) 3D modelling of the brain of *Tg(−3.1ngn1:GFP*) zfl on Tfap2e KD at 2 days post fertilisation (dpf) and box plot graph of the forebrain hemisphere cleft measured as indicated (white brackets). (**A**) shows a schematic depiction of zfl brain in transverse (left) and coronary view (right) (modified from Dworschak *et al*, 2021).^28^ Pink: olfactory blub. Turquoise: mesencephalon. Purple: rhombencephalon. (**B**) The representative images were obtained by in vivo 2-photon z–stack imaging and subsequent modulation with IMARIS software. Transverse (left) and coronary view (right). (**C**) TB MO and e3i3 morphant zfl present with an enlarged hemisphere cleft consistent with a hydrocephalus. Note that only TB MO but not e3i3 MO treated zfl show a reduced brain volume (see online supplemental data 13). (**D–E**) Representative confocal in vivo images of *Tg(−3.1ngn1:GFP*) zfl at 2 dpf in transverse view after sulforhodamine SR101 injection (in red) into the diencephalic ventricle and box plot graph of ventricle diameters (white brackets) in relation to hemisphere diameter (dotted line). In TB MO zfl, both the diencephalic and the rhombencephalic ventricle are enlarged in the sense of a hydrocephalus. ***p<0.001, ****p<0.0001 (C: one-way ANOVA with Tukey’s multiple comparison; E: two-way ANOVA with Tukey’s multiple comparison). Number of independent experiments n=3 for all graphs, number of investigated zfl *n* as indicated. White scale bars: 200 µm. ANOVA, analysis of variance; DV, diencephalic ventricle; KD, knockdown; MO, morpholino oligonucleotide; ns, not significant; RV, rhombencephalic ventricle; TB, translation blocking; TV telencephalic ventricle; zfl, zebrafish larvae.

We further evaluated the CNS phenotype by in vivo 2-photon microscopy in *Tg(−3.1.ngn1:GFP*) KD zfl at 2 dpf. Therefore, we created a high-resolution 3D brain model from z-stack images of zfl treated with Ctrl MO, TB MO or e3i3 MO (figure 4A,B, online supplemental data 9, onlinesupplemental videos 1^3^). TB MO zfl presented with a significantly reduced brain volume (2.1 *10^6^ µm³) compared with controls (3.6 *10^6^ µm³). Interestingly, the brain volume of e3i3 MO treated zfl (3.2 *10^6^ µm³) was not significantly reduced (online supplemental data 13). Furthermore, we could detect an obvious hydrocephalus visible through an enlarged forebrain hemisphere cleft in TB MO and e3i3 MO zfl (44 µm; 57 µm; Ctrl MO 19 µm) (figure 4A–C, onlinesupplemental videos 1^3^). To obtain further evidence that this hemisphere cleft is not a sign of a local structural malformation but of a general hydrocephalus, sulforhodamine 101 (SR101) injections were performed. In TB MO zfl an increased diameter of the rhombencephalic ventricle (2.4; Ctrl MO 1.7) and diencephalic ventricle (0.7; Ctrl MO 0.3) was observed (figure 4D,E). These findings were supported in a *tfap2e* KO^CRISPR^ line crossed into *Tg(−3.1ngn1:GFP*), showing a mild hydrocephalus in *tfap2e*^+/-^ and *tfap2e*^-/-^ zfl compared with wt littermates (online supplemental data 14). However, unlike Tfap2e KD zfl, KO^CRISPR^ larvae did not exhibit a severe phenotype in brightfield imaging and only a subset of *tfap2e*^+/-^ or *tfap2e*^-/-^ zfl presented with variable hydrocephalus.

## Discussion

*TFAP2E* has not yet been described in the aetiology of human NDD or congenital anomalies. The individuals described here with heterozygous missense variants in the neural crest specifier *TFAP2E* showed a phenotypical spectrum of CNS, orofacial and maxillofacial anomalies that are associated with neural crest and neural plate border specification failures.^30 31^ Shared phenotypes of the seven individuals from five families comprise orofacial and maxillofacial anomalies as the most frequent phenotype (6/7), CNS anomalies (4/7) and NDD (2/5). All of these features are reminiscent of neurocristopathies. Interestingly, a well-characterised neurocristopathy, branchio-oculo-facial syndrome (MIM: 113620), is caused by heterozygous missense variants in the paralog *TFAP2A*.^32^ In a zebrafish model of melanophore differentiation a redundancy of Tfap2a and Tfap2e was shown, suggesting functional similarities. Here, we report for the first time the implication of TFAP2E in human congenital anomalies.

The finding of the c.337C>A *TFAP2E* variant segregating in affected family members over three generations (family B) provides strong evidence for our supposed implication of heterozygous *TFAP2E* variants with CNS, orofacial and maxillofacial anomalies. However, individual B-II:2 is also heterozygous for the c.337C>A variant but has no history of congenital CNS, orofacial and maxillofacial anomalies. Her neurological phenotype (ie, migraine and anxiety disorder) was not described in other individuals with *TFAP2E* variants and could likely be of non-genetic origin. Although we cannot provide evidence, we assume reduced penetrance for the c.337C>A variant in individual B-II:2. In fact, reduced penetrance is well described for many severe developmental defects and neurocristopathies, such as holoprosencephaly type 3 (MIM: 142945) or Waardenburg syndrome type 3 a (MIM: 277580). Additional human genetic evidence is provided by the de novo occurrence of a novel *TFPA2E* variant (c.682C>T) in one family (family D). Unfortunately, we could not assess for de novo occurrence in the other three families (families A, C and E). Nevertheless, in silico analysis of all variants predict a damaging effect on TFAP2E (figure 1G, table 2). Further support is given by our protein structure modelling (online supplemental data 6, online supplemental table 1).

Interestingly, 18 individuals with CNVs overlapping the *TFAP2E* locus ranging from 0.5 Mb to 16.7 Mb listed in DECIPHER show phenotypical similarities to the observed features in the individuals reported here with single nucleotide variants in *TFAP2E*, that is, NDD and craniofacial anomalies. Of course, due to the large size of these CNVs and the predicted tolerance to loss of function (pLI=0), the contribution of these CNVs to the observed phenotypes remains speculative (online supplemental table 2).

Our zebrafish data showed that KD of Tfap2e in zfl has multiple effects on NCC after neural plate border specification.^13^ Orofacial and maxillofacial structures are known to be highly dependent on NCCs that give rise to cartilage cells, that is, in the region of the anterior arches. The developmental stage of the zfl at 3 dpf is also called the ‘protruding-mouth stage’ as orofacial and maxillofacial development is promoted at this stage and zfl develop an open mouth.^33^ We show here that Tfap2e is significantly upregulated at this developmental stage (figure 2A). Strikingly, these structures are hypoplastic and severely malformed in the Tfap2e KD zfl, as demonstrated by Alcian cartilage staining (figure 3C–E).

Moreover, the neural crest specifier Sox10 is needed for the formation of anterior and branchial arches of the craniofacial skeleton.^34^ Sox10 expression has been postulated to be promoted by Tfap2e via Snail2.^9^ Following the Tfap2e KD, we show disrupted sox10:mRFP reporter-signal in the region of the anterior and branchial arches (figure 3A,B). Previous reports of Sox10 deficiency in zfl further described that *ngn1* transcription is not induced. This lack in *ngn1* then led to a reduction in the number of DRG neurons in the developing spinal cord.^35^ In a similar manner, our in vivo microscopy analysis of zfl after Tfap2e KD revealed a specific reduction of DRG neurons (figure 3F,G), but not of RBSN, the progenitor cell line of DRG neurons (online supplemental data 12). Taken together, this indicates that Tfap2e KD is responsible for incorrect replacement of RBSN by DRG neurons through Sox10 deficiency.^36 37^ The interaction of human *SOX10* and *TFAP2E*, however, needs to be further studied. Features such as hearing impairment and early hair greying that are known to occur within the *SOX10*-deficiency associated Waardenburg syndrome (MIM: 611584) were not found in the individuals reported here with *TFAP2E* variants.

Our protein expression data indicate precise temporal and spatial regulation of Tfap2e in early larval development (figure 2A,B). This dynamically regulated expression pattern very likely explains the failure to rescue the morphant phenotype by co-injection of unregulatable human or zf mRNA. Accordingly, zfl co-treated with Ctrl MO and wt *TFAP2E* human mRNA showed a mild, morphant-like phenotype, indicating that unregulated overexpression of TFAP2E is also disruptive (figure 2E).

The two different MO KD strategies (ie, translation and splice blocking) and the KO^CRISPR^ model, show obvious phenotypical overlap, indicating a specific effect of Tfap2e deficiency. Specifically, the TB MO also targets maternal RNA and the KO^CRISPR^ excludes p53-mediated effects. However, limitation arises as further analysis of the MO specificity and functional consequences of the specific human *TFAP2E* variants was not possible due to the aforementioned failure of rescue observed in the mRNA experiments. Thus, we also cannot assess the pathogenicity of the human variants in our zf model. Additionally, the orthologous amino acids of two of the five human variants identified here are not conserved in zebrafish (table 2), which makes it impossible to functionally assess them in this model. Testing these variants in mice, where all five variant residues are conserved and the TFAP2E homology is 93%, compared with 77% in zebrafish, could provide solid evidence of pathogenicity for specific variants.

Remarkably, hydrocephalus was present in four of seven individuals with variants in *TFAP2E* and was recapitulated in our zfl KD and KO^CRISPR^ models. Previously, van Otterloo *et al*^14^ noticed opaque areas in the hindbrain following injection of the e3i3 MO. Van Otterloo *et al* considered this not as a specific CNS phenotype but as non-specific toxicity resulting in apoptosis. Although our CNS-specific analysis in Tfap2e KD zfl showed a reduction in brain volume in the TB MO-treated zfl, we did not observe this phenotype in the e3i3 MO-treated zfl (online supplemental data 13). Regardless of the differing brain size, we found hydrocephalus in both MO groups. Thus, we postulate that hydrocephalus represents a specific effect following Tfap2e KD. Remarkably, we also observed this most-penetrant human phenotype in both, *tfap2e*^+/-^ and *tfap2e*^-/-^ KO^CRISPR^ zfl, underpinning the role of *tfap2e* in congenital hydrocephalus (online supplemental data 14). Although, the hydrocephalus only displayed in a subset of KO^CRISPR^ zfl, indicating incomplete phenotypical penetrance, it nonetheless reinforces the involvement of *tfap2e* in congenital hydrocephalus. It also resembles reduced penetrance in humans as seen in family B.

Of note, the parents of D-II:1 had in a later pregnancy a fetus (D-II:4) with partial phenotypical overlap. However, this later fetus did not carry the *TFAP2E de novo* variant detected in D-II:1. ES did not identify any other disease-attributable variant in the later fetus (D-II:4). Furthermore, there was no independent genetic factor in individual D-II:4. Since D-II:4 presented with cerebellar hypoplasia and retrognathia only but did not show hydrocephalus, microlissencephaly, thinned brain stem nor hypotrophic basal ganglia, we suspect potentially distinct and independent entities. Given the strong genetic evidence by a novel de novo variant in *TFAP2E* and the phenocopy in our zebrafish model with hydrocephalus and brain phenotype, we see solid evidence that *TFAP2E* is implicated in the phenotype of D-II:1.

Given the overall tolerance of *TFAP2E* to loss-of-function variants (pLI=0), other disease mechanisms should be considered for humans compared with the hypothesis of a dosage-dependent effect in our zf KD/overexpression model. Impairment of correct heterodimerisation has been previously established as a pathomechanism for TFAP2E paralog proteins TFAP2A-C.^7^ Likewise, the human variant residing in the dimerisation domain in E-II:1 (p.Gly312Ser) might impair correct dimerisation. However, we can only speculate whether the human variants presented here confer a dominant negative effect, as our variant analysis is limited to in silico predictions. To conclusively establish the disruptive function of the investigated *TFAP2E* variants presented here and rule out the possibility of non-specific observations, further studies are needed. These could include in vitro models or knock in animal models allowing for an accurate assessment of impaired variant functionality.

Nonetheless, the presented human genetic and embryonic zebrafish data suggest a potential role of *TFAP2E* in aberrant CNS, orofacial and maxillofacial development.

## supplementary material

10.1136/jmg-2023-109799 online supplemental file 1

10.1136/jmg-2023-109799 online supplemental file 2

10.1136/jmg-2023-109799 online supplemental table 1

10.1136/jmg-2023-109799 online supplemental table 2

10.1136/jmg-2023-109799 online supplemental file 3

10.1136/jmg-2023-109799 online supplemental file 4

10.1136/jmg-2023-109799 online supplemental file 5

## Data Availability

All data relevant to the study are included in the article or uploaded as supplementary information.
