# Supplementary material for: TFAP2E is implicated in central nervous system, orofacial and maxillofacial anomalies
Source: J Med Genet. 2024 Dec 23;62(2):e109799. doi: 10.1136/jmg-2023-109799 (PMC11777392; doi:10.1136/jmg-2023-109799)
Supplement: online supplemental file 1 [file jmg-62-2-s001.pdf]

Supplementary Material

TFAP2E is implicated in central nervous system, oro- and maxillofacial anomalies

Jeshurun C. Kalanithy,[1,c,*] Enrico Mingardo,[1,3] Jil D. Stegmann,[1,3] Ramgopal Dakar,[4] Tikam C. Dakal,[4] Jill A. Rosenfeld,[5,6] Wen-Hann Tan,[7] Stephanie A. Coury,[7,8] Audrey C. Woerner,[9] Jessica Sebastian,[9] Paul A. Levy,[10] Leah R Fleming,[11] Lea Waffenschmidt,[1] Tobias T Lindenberg,[2] Öznur Yilmaz,[2] Khadija Channab,[3] Bimaljeet Kaur Babra,[2] Andrea Christ,[3] Britta Eiberger,[3] Selina Hölzel,[1,2] Clara Vidic,[1] Felix Häberlein,[3,12] Nina Ishorst,[1,2] Juan E. Rodriguez-Gatica,[13] Behnaz Pezeshkpoor,[14,15] Patrick A. Kupczyk,[16] Olivier Vanakker,[17] Sara Loddo,[18] Antonio Novelli,[18] Maria L. Dentici,[19] Albert Becker,[20] Holger Thiele,[21] Jennifer E. Posey,[5] James R. Lupski,[5,22,23] Alina C. Hilger,[24,25] Heiko Reutter,[1,26,27] Waltraut M. Merz,[28†] Gabriel C. Dworschak,[1,2,29†] and Benjamin Odermatt,[2,3†*].

***Correspondence to:**

Jeshurun Chiaran Kalanithy, Institute of Neuroanatomy, Medical Faculty, University of Bonn, Bonn 53115, Germany (jkalanithy@uni-bonn.de, Tel.: +49 (0)228 739033)

Benjamin Odermatt, Institute of Neuroanatomy, Medical Faculty, University of Bonn, Bonn 53115, Germany (b.odermatt@uni-bonn.de, Tel.: +49 (0)228 739033)

1 Supplementary Files

Supplementary Table 1: Molecular data of individuals with CNVs encompassing *TFAP2E* and available phenotype data.

Supplementary Table 2: Phenotype data of individuals with heterozygous variants and copy number variations encompassing *TFAP2E*.

Supplementary Data 1: Detailed clinical and genetic reports of human subjects

Individual A-II:1 derives of consanguineous parents and presents with a Chiari II malformation with thoracolumbar myelomeningocele, hydrocephalus treated with a ventriculoperitoneal shunt, partial agenesis of corpus callosum, ID/DD, paraplegia, hypotonia, neurogenic bladder, micrognathia, vertebral body segmentation defects, multiple rib fusions, hypoplastic 5th toe nails and persistent digit pads of fingers and toes (FIGURE 1A, TABLE 2, Supplementary Table 2). Exome sequencing revealed a novel heterozygous missense variant c.23C>A (p.Ala8Asp) in *TFAP2E*, residing in a promoter region of exon 1 (Zerbino et al., 2015). *In silico* prediction tools rated the variant to be deleterious (TABLE 1). Parental DNA for segregation was not available.

Individual B-III:1 presented postnataly with enlarged bifrontal extra-axial fluid collections in the sense of external hydrocephalus and choroid plexus cysts (FIGURE 1A, TABLE 2, Supplementary Table 2). Additionally, he presented with retro- and micrognathia, cleft lip and palate, low-set ears, abnormal nasal bridge, short nose, deep set eyes, downslanting palpebral fissures, overlapping toes, single transverse palmar crease, cardiac anomalies (patent ductus arteriosus, patent foramen ovale, ventricular septal defect), congenital diaphragm hernia and capillary haemangioma. Exome sequencing revealed a novel heterozygous missense variant c.337C>A (p.Pro113Thr) in *TFAP2E*, residing in a promoter region of exon 2. *In silico* prediction tools rated the variant to be tolerated or possibly damaging (TABLE 1). Sanger sequencing of the parents confirmed the variant to be inherited by the mother (B-II:2) and carrier status of the uncle (B-II:4) and grandfather (B-I:2). Of note, the maternal family history is indicative of a dominantly inherited disease of reduced penetrance. The maternal grandfather B-I:2 presented with cleft palate and scoliosis. A maternal uncle B-II:4 presented with cleft palate, scoliosis and ptosis. A maternal aunt B-II:3 had a learning disability. The mother B-II:2 did not present within the phenotypic spectrum; she had a history of migraine and an anxiety disorder. Mother's paternal half-brother B-II:5, who is the child of B-I:2, presented with intellectual disability.

Individual C-II:1 presented with a thalamic lesion, intellectual disability and developmental delay, attention–deficit/hyperactivity disorder, macrocephaly, tall stature, advanced bone age, obesity and fatty liver (FIGURE 1A, TABLE 2, Supplementary Table 2). Exome sequencing revealed a novel heterozygous missense variant c.386G>A (p.Arg129His) in *TFAP2E*, residing in a promoter region of exon 2. *In silico* prediction tools rated the variant to be tolerated or probably damaging (TABLE 1). Parental DNA was not available for segregation.

Individual D-II:1 was prenatally diagnosed with microlissencephaly, hypoplastic cerebellum, hydrocephalus (enlarged external cerebrospinal fluid space), thinned brain stem, hypotrophic basal ganglia, retrognathia (FIGURE 1B-E, TABLE 2, Supplementary Table 2). Termination of pregnancy was performed on maternal indication. Family exome sequencing revealed a novel *de novo* missense variant c.682C>T (p.Leu228Phe) in *TFAP2E* in D-II:1. *In silico* prediction tools rated the variant to be deleterious (TABLE 1). Subsequently, the same couple had another pregnancy with a male fetus (D-II:4) presenting with cerebellar hypoplasia only and retrognathia. However, exome sequencing of D-II:4 did not reveal a variant in *TFAP2E*. Allele-specific PCR did not reveal a mosaic variant of the *TFAP2E* c.682C>T variant in the parental DNA sample extracted from blood sample. There were no other *de novo* variants in neurodevelopmental disorder disease genes detected in D-II:1 or D-II:4. Likewise, no common deleterious autosomal recessive variants in genes involved in CNS development were detected. We detected one missense variant in *OPHN1* (c.410A>G, p.Asp137Gly) in D-I:2, D-II:1 and D-II:4. Loss-of-function variants in *OPHN1* are associated with X-linked recessive intellectual disability and cerebellar hypoplasia. We tested the family for X-inactivation using the HUMARA assay (data not shown). We were able to show that the maternal allele harbouring the *OPHN1* variant is skewed in the maternal blood cells (82.8 % activity), while the female fetus D-II:1 shows a balanced

activation of 57.9 %. We therefore rated this variant as likely benign as the mother was asymptomatic although the variant allele showed more activity than D-II:1.

Individual E-II:1 is a male individual of latin american ethnicity presenting with alobar holoprosencephaly, hydrocephalus, macrocephaly, cleft lip and palate, low-set ears, abnormal nasal bridge, short nose, hypertelorism, craniotabes, congenital scoliosis, cardiac anomalies, prematurity, pectus excavatum, anasarca and solitary kidney (FIGURE 1A, TABLE 2, Supplementary Table 2). Exome Sequencing detected an ultra-rare heterozygous missense variant (MAF 0.00003 in Latino/admixed American population) in *TFAP2E* c.934G>A p.(Gly312Ser). *In silico* prediction tools rated the variant to be deleterious (TABLE 1). Parental DNA for segregation was not available.

References: Zerbino, D. R., Wilder, S. P., Johnson, N., Juettemann, T., and Flicek, P. R. (2015). The ensembl regulatory build. *Genome Biol* 16. doi: 10.1186/S13059-015-0621-5.

S2

A

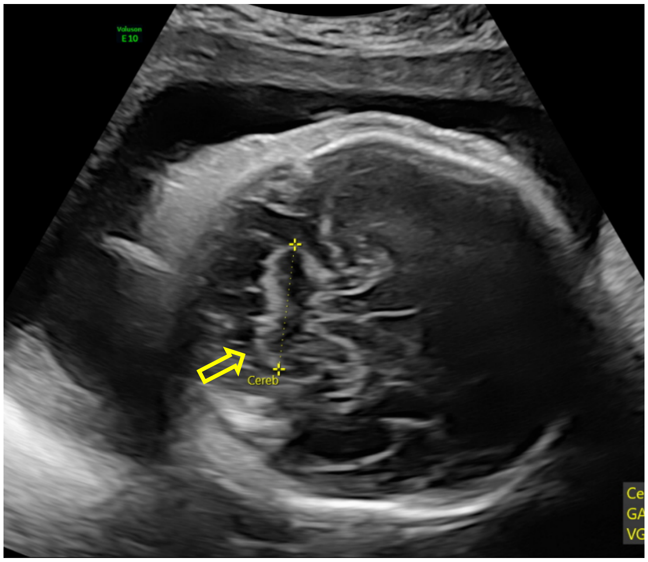

B

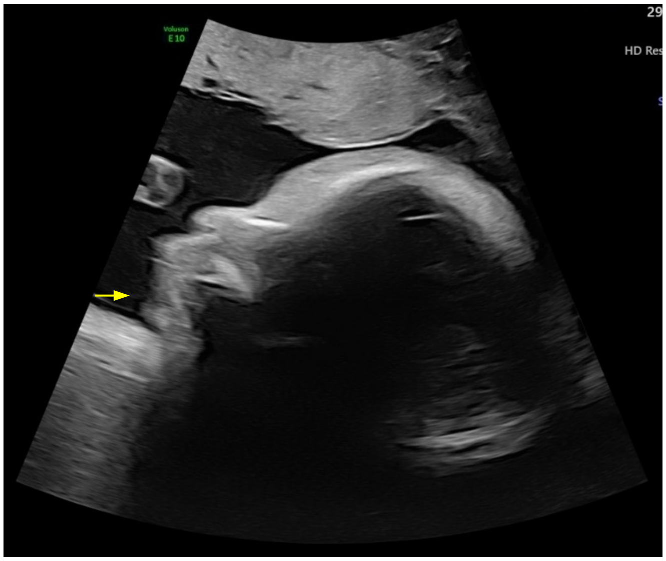

Supplementary Data 2: Prenatal ultrasound imaging of fetus D-II:4

(A-B): Affected fetus D-II:4, 31+2 weeks of gestation. Ultrasound examination, (A) transverse plane showing cerebellar hypoplasia (yellow hollow arrow) (transverse cerebellar diameter: 25.3 mm. Reference range, 5. – 95. percentile: 32.0 mm – 44.9 mm; Donadono et al., 2021) (B) sagittal plane showing retrognathia (yellow arrow).

References: Donadono V, Cavallaro A, Roberts NW, Ioannou C, Papageorghiou AT, Napolitano R. A Systematic Review of Methodology Used in Studies Aimed at Creating Charts of Fetal Brain Structures. Diagnostics (Basel). 2021 May 21;11(6):916. doi: 10.3390/diagnostics11060916.

Suppplementary Data 3: Copy number variations encompassing *TFAP2E* present with neurodevelopmental disorders and craniofacial anomalies

We further reviewed the DECIPHER database[1], Baylor Genetics clinical chromosomal microarray database and conveyed literature research for missense variants in *TFAP2E* and copy number variations (CNVs) encompassing *TFAP2E* in 1p34.3. We did not identify any additional individuals with missense variants, but 24 individuals with CNVs (Firth et al., 2009, Galesi et al., 2022, Tokita et al., 2015). Of those, 18 individuals presented with a deletion and six individuals with a duplication encompassing *TFAP2E* (Supplementary Table 1, Supplementary Data 4). Eighteen individuals had available phenotype data. Of these CNVs, 56 % (10/18) were confirmed to occur *de novo* and six percent (1/18) as *de novo* mosaic. One CNV (6 %) was maternally inherited, the remaining 33 % (6/18) were of unknown inheritance. Size of CNVs varied from 0.5 Mb to 16.7 Mb. Shared phenotypes comprised craniofacial anomalies (15/18, 83 %) including pro- or retrognathia (8/18, 44 %), abnormal nasal bridge (7/18, 39 %), ear anomalies (7/14, 50 %), frontal bossing (5/18, 28 %), high arched palate (3/18, 17 %) or macrocephaly (3/18, 17 %). Neurodevelopmental phenotypes included intellectual disability / developmental delay (12/18, 71 %), hypotonia (9/18, 43 %), attention-deficit/hyperactivity disorder (5/18, 29 %), and hydrocephalus (1/14, 7 %) (Supplementary Table 2). We defined a minimum region of overlap of the CNVs between chr1:35552211-chr1:35909938 (GRCh38) encompassing eight genes including *KIAA0319L*, *NCDN, TFAP2E, PSMB2, C1orf216, CLSPN, AGO4* and *AGO1* (Supplementary Data 4). Of those, only *NCDN* has previously been described in the context of neurodevelopmental disorders with intellectual disability / developmental delay (MIM #619373) in individuals with biallelic or *de novo* heterozygous *NCDN* variants (Fatima et al., 2021). Other key phenotypic features as microcephaly (2/18, 11 %) or infantile epileptic spasms (0/18) are not represented accordingly in this cohort and therefore limit evidence for a contribution of *NCDN* to the phenotypic spectrum. In comparison to individuals with heterozygous *TFAP2E* variants, the dominance of the craniofacial phenotype is equally represented (15/18, 83 %). Neurodevelopmental findings only include structural brain anomalies in one individual, thus indicating a milder phenotype. However, incomplete phenotype records cannot be excluded for the other cases. We conclude that dosage-sensitivity of *TFAP2E* might contribute to the phenotypic expression of the presented cases.

1. Firth, H.V., Richards, S.M., Bevan, A.P., Clayton, S., Corpas, M., Rajan, D., van Vooren, S., Moreau, Y., Pettett, R.M., and Carter, N.P. (2009). DECIPHER: Database of Chromosomal Imbalance and Phenotype in Humans Using Ensembl Resources. American journal of human genetics *84,* 524–533

S4

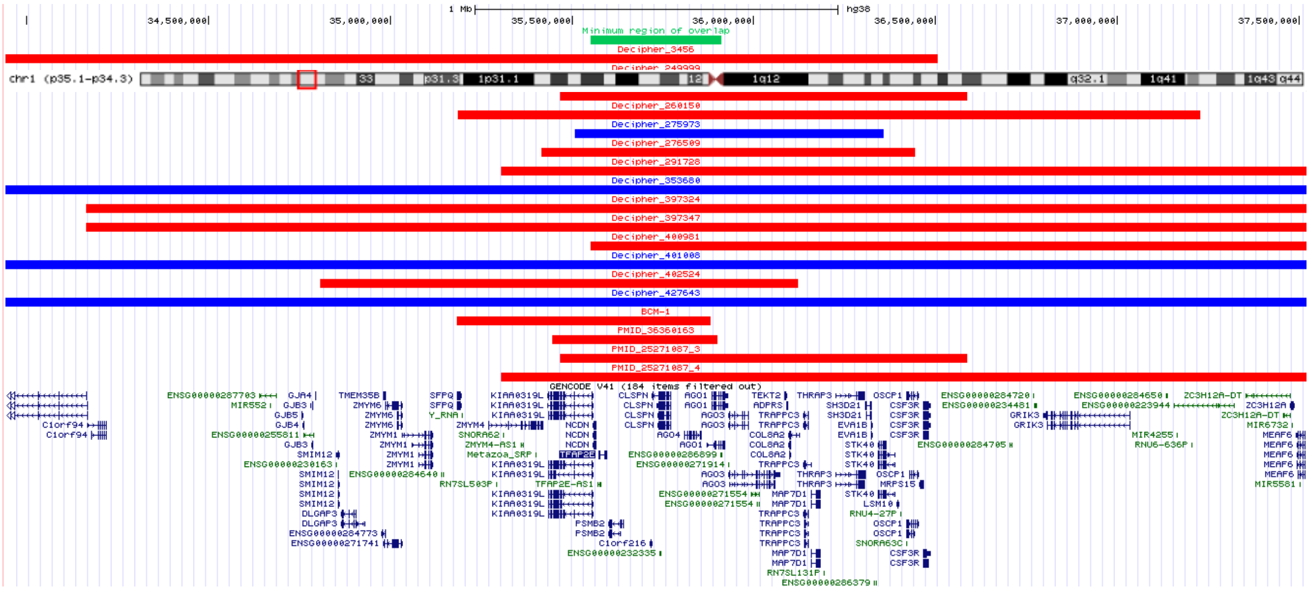

Supplementary Data 4: Genomic location of copy number variations encompassing *TFAP2E*

Visualization of chromosome 1 and the region of copy number variations (upper panel, red rectangle) encompassing *TFAP2E*. Lower panel: Screenshot of UCSC web browser at chromosome region 1p34.3 and encompassing deletions (red) and duplications (blue). Green UCSC track: Minimum region of overlap of all assessed CNVs. Genes located in this region are *KIAA0319L*, *NCDN, TFAP2E, PSMB2, C1orf216, CLSPN, AGO4* and *AGO1*.

Supplementary Data 5: Detailed description of 3D protein modeling of heterozygous TFAP2E variants

The genomic and the aa sequence of TFAP2E were retrieved from NCBI (https://www.ncbi.nlm.nih.gov) and Uniprot (https://www.uniprot.org), respectively. The identified human *TFAP2E* variants were mapped accordingly on the wt TFAP2E protein sequence. The 3D structure prediction of the TFAP2E protein was done by using the RaptorX web server (http://raptorx.uchicago.edu/ContactMap/), which predicts a total of five models for each variant. The model which showed the lowest root–mean–square-deviation (RMSD) value was selected for further analysis. In addition, the structure modelling of TFAP2E protein variants was done by using the Chimera software v. 1.14 (Pettersen et al., 2004). For structural comparison, superimposition of TFAP2E protein variants onto the wt TFAP2E protein was done by using the MatchMaker tool, present in the Chimera software.

Individual	Variant	RMSD (Å)
	TFAP2E_Wild	12.757
A-II:1	TFAP2E_Variant_1	12.342
B-III:1	TFAP2E_ Variant_2	12.444
C-II:1	TFAP2E_ Variant_3	13.526
D-II:1	TFAP2E_ Variant_4	12.667
E-II:1	TFAP2E_ Variant_5	13.428

Screenshots of the 3D protein modelling are provided in Supplementary Data 6.

Individual A-II:1:

In TFAP2E_Variant_1 c.23C>A; p. Ala8Asp. The amino acid (aa) sequence was altered to p.Ala8Asp. In the structural modelling the Ala (Non-polar) and Asp (Polar) generates the same loop structure, but this mutation causes the other aa to modify their secondary structure formation, helix to loop or vice versa, and sheet to loop or vice versa. Change in the polarity of the aa may increase the hydrophilicity of the protein. Due to polarity change the conformation of the protein also changes, and the function of nearby aa may be altered or the whole protein deactivated.

The following table shows the nearest secondary structure modification in wild and mutant TFAP2E protein.

S. No.	Position	The amino acid in wild variant with acquired structure	The amino acid in mutant variant_1 with acquired structure
1	8	A – Loop	D - Loop
2	47-49	AAE - Helix	AAE - Loop

Individual B-III:1:

The amino acid sequence of TFAP2E protein from 22 to 122 forms the transactivation domain. Protein scaffold domain that has a binding site for other proteins. In TFAP2E_Variant_2 c.337C>A; p. Pro113Thr. The aa sequence was altered to p.Pro113Thr. In the structural modelling the Pro (Non-polar) and Thr (Polar) generates the same loop structure, but this mutation causes the other aa to modify their secondary structure formation, helix to loop or vice versa, and sheet to loop or vice versa. Change in the polarity of the aa may increase the hydrophilicity of the protein. Due to polarity change the conformation of the protein also changes, and the function of nearby aa may be altered or deactivate the whole protein. Alteration in Pro113Thr may alter or deactivate the transactivation domain function.

The following table shows the nearest secondary structure modification in wild and mutant TFAP2E protein.

S. No.	Position	The amino acid in wild variant with acquired structure	The amino acid in mutant variant_2 with acquired structure
1	50	F - Loop	F - Helix
2	96-99	PQAA - Helix	PQAA - Loop
3	113	P – Loop	T - Loop
4	123-126	ALGL - Loop	ALGL - Helix
5	128-130	PRR - Loop	PRR - Helix

Individual C-II:1:

In TFAP2E_Variant_3 c.386G>A; p. Arg129His. The aa sequence was altered to p.Arg129His. In the structural modelling the Arg and His generates the same loop structure, but this mutation causes the other aa to modify their secondary structure formation, helix to loop or vice versa, and sheet to loop or vice versa.

The following table shows the nearest secondary structure modification in wild and mutant TFAP2E protein.

S. No.	Position	The amino acid in wild variant with acquired structure	The amino acid in mutant variant_3 with acquired structure
1	120-124	PARAL - Loop	PARAL - Helix
2	129	R – Loop	H - loop
3	134	T - Loop	T - Helix

Individual D-II:1:

In TFAP2E_Variant_4 c.682C>T; p. Leu228Phe. The aa sequence was altered to p.Leu228Phe. In the structural modelling the Leu and Phe generate the same loop structure, but this mutation causes the

other aa to modify their secondary structure formation, helix to loop or vice versa and sheet to loop or vice versa.

The following table shows the nearest secondary structure modification in wild and mutant TFAP2E protein.

S. No.	Position	The amino acid in wild variant with acquired structure	The amino acid in mutant variant_4 with acquired structure
1	222	P - Sheet	P - Loop
2	228	L – Loop	F - Loop
3	233	K - Sheet	K - Loop
4	245	L - Loop	L - Helix

Individual E-II:1:

In TFAP2E_Variant_5 c.934G>A; p. Gly312Ser. The amino acid sequence was altered p.Gly312Ser. In the structural modelling the Gly (Non-polar) and Ser (Polar) generates the same helix structure, but this mutation causes the other aa to modify their secondary structure formation, helix to loop or vice versa, and sheet to loop or vice versa. Change in the polarity of the amino acid may increase the hydrophilicity of the protein, due to polarity change the conformation of the protein also changes, and the function of nearby aa may be altered or deactivate the whole protein.

The following table shows the nearest secondary structure modification in wild and mutant TFAP2E protein.

S. No.	Position	The amino acid in wild variant with acquired structure	The amino acid in mutant variant_5 with acquired structure
1	294	T - Helix	T - Loop
2	312	G – Helix	S - Helix
3	332	H - Helix	H - Loop

Supplementary Data 6: Screenshots of 3D protein modelling of TFAP2E missense variants

See additional file. 3D protein modelling of human monoallelic TFAP2E variants, as indicated, using described methods. The wildtype conformation is indicated in green, the individual's variation is indicated in red. The structural variations are described in detail in Supplementary Data 5.

S7

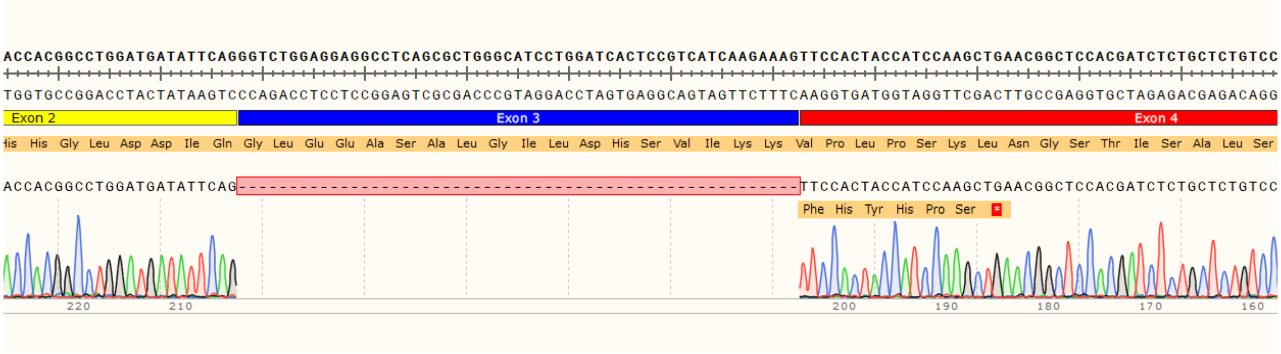

Supplementary Data 7: Tfap2e knockdown with e3i3 Morpholino leads to precise exclusion of exon 3

Upper panel: Snapgene screenshots of *Danio rerio* tfap2e reference sequence with annotation of exons 2-4 and amino acid code. Lower panel: Alignment of the Sanger sequenced PCR product (second row) after rtPCR against *tfap2e* in zebrafish larvae treated with e3i3 MO. This MO leads to precise exon skipping of exon 3, resulting in a frameshift and consequently a premature stop codon after six amino acids.

Supplementary Data 8: List of commercially obtained reagents

Reagent	Distributor
pCMV-Sport6.1-based vector with human wt *TFAP2E* (ENST00000373235.4)	Source BioScience IMAGE ID 5786430
pCMV-Sport6.1-based vector with the zebrafish wt *tfap2e* (ENSDART00000190005.1)	Source BioScience IMAGE ID 6800961
mMESSAGE mMACHINE™ SP6 Ultra Transcription Kit	ThermoFisher Invitrogen™, Catalog No. AM1340
Invitrogen™ Poly-(A) Tailing Kit	ThermoFisher, Invitrogen™, Catalog No. AM1350
Anti-Tfap2e antibody	Thermofisher, Catalog No. PA5-72631
iScript™ cDNA Synthesis Kit	Bio-Rad, Catalog No. 170889

Supplementary Data 9: Detailed imaging methods and phenotyping of transgenic zebrafish larvae

For evaluation of the CNS phenotype, we *in vivo* imaged the brain (from dorsal) and the spinal cord (from lateral) of *Tg(-3.1ngn1:GFP)* morphants using a 2-photon microscope equipped with a scan head based laser-scanning microscope TriM Scope™ (LaVision, BioTec, Bielefeld, Germany), a 20x water-immersion objective lens (NA1.0, W Plan-Apochromat, Zeiss) and tuneable TiSa laser (InSigh Deepse, Spectra-Physics, Santa Clara, USA) (Häberlein et al., 2022). We aquired z-stack images with 2 µm step size. Image processing, analyses and 3D representation of the data were facilitated through IMARIS (Version 9.5.1., Oxford Instruments plc) running on a workstation equipped with two Intel Xeon Platinum 8160 CPU (2.1 GHz, 24 cores), 512 GB memory, and an Nvidia Quadro P5000 GPU (16 GB GDDR5X) running under Windows 10 Pro. Briefly, we created a workflow including (i) selection of representative region of interest (ROI) within the brain, (ii) manual lower thresholding, (iii) computed selection of voxels belonging to the CNS (iv) manual deselection of larger artifacts (v) application to whole brain. The created object was analyzed regarding its volume for the microencephaly phenotype. Moreover, we measured the cleft with the forebrain hemispheres. Additionally, we used the 3D model to manually count RBSN, DRG neurons and outgrowing central and peripheral projecting axons in the starting segment of the spinal cord over a distance of 500 µm.

For evaluation of the hydrocephalus phenotype, we performed *in vivo* microinjections with 1.7 nl of 20 µM sulforhodamine 101 (SR101, Catalog No. S359) into the diencephalic ventricle of *Tg(-3.1ngn1:GFP)* zfl at two dpf and successive imaging with a Nikon A1 R HD25 Ti2-E confocal laser scanning microscope using a 20x oil-immersion objective lens (NA 0.95). Injection techniques were adapted as previously described (Lowery and Sive, 2005). We captured z-stack images with 2 µm step size and measured the broadest diameter of the rhombencephalic, diencephalic and telencephalic ventricles in TB MO treated zfl and respective controls. To account for variability in brain size, these measurements were then put in relation to the diameter of the right brain hemisphere.

Alcian Blue Cartilage staining was performed with zfl at three dpf using predescribed methods (Walker and Kimmel, 2007). The white brackets (FIGURE 3C) show the measured distance from the rostral end of the Meckel ‘s cartilage to the caudal point of the hyosympletic cartilage.

Evaluation of NCC distribution in craniofacial structures at three dpf was performed in *Tg(sox10:mRFP)* outcrossed with wt AB/TL strain using a ZEISS Axio Zoom.V16 and subsequent fluorescence intensity analysis using Fiji ImageJ. For imaging, the microscope was focused on the lower jaw region of zfl, using the same light intensity and filter settings (EX: 587/25, BS: 605, EM: 647/70), exposure time, zoom and further microscope parameters for morphants and controls. The square region of interest was defined as the complete head region excluding the fins and the yolk, and measured as sox10:mRFP fluorescence signal per µm^2.

S10

```
zebrafishTfap2e      MLVHSYSSMERADGLSSSSPGGRLSQLSQLNQAAYSSAPPLCHTPA----SDFQPPYFPP    56
humanTFAP2E          MLVHTYSAMERPDGLGAAAGGARLS---SLPQAAYGPAPPLCHTPAATAAAEFQPPYFPP    57
mouseTFAP2E          MLVHTYSAMERPDGLGAAAGGTRLS---SLPQAAYGPAPPLCHTPAASATADYHPPYFPP    57
                     ****:**:*** ***.::: * ***   .* ****. *********    ::::******

zebrafishTfap2e      PYPQSSLSYSQSQDG--GYPHLP-EPYPSLNSLHQ--HQQAAWHSQ----RSRSEDAGLL    107
humanTFAP2E          PYPQPPLPYGQAPDAAAAFPHLAGDPYGGLAPLAQPQPPQAAWAAPRAAARAHEEPPGLL    117
mouseTFAP2E          PYPQAPLPYGQGPDATAAFPHLAADPYGGLAPLAQPQPPQAAWAAPRAAARAHDEPPGLL    117
                     ****  * *.*. *.  .:***  :** .*  * *    **** :     *::.*  ***

zebrafishTfap2e      SQPHRALSLDPRREYPGVPRLLTHGLGDGAAALGDGPLGMHAV--HHGLDDIQGLEEASA    165
humanTFAP2E          APPARALGLDPRRDYATAVPRLLHGLADGAHGLADAPLGLPGLAAAPGLEDLQAMDE-PG    176
mouseTFAP2E          APPARALGLDPRRDYAAAVPRLLHSLADGAHGLADAPLGLPGLAEPPGLEELQAIDD-PG    176
                     : * ***.*****:*  .   * *.*.*** .*.*.***: .:    **:::*.:::  .

zebrafishTfap2e      LGILDHSVIKKVPLPSKLNGSTISALSLSKEGLGLGGVSNPAEVFCSVPGRLSLLSSTSK    225
humanTFAP2E          MSLLDQSVIKKVPIPSKAS--SLSALSLAKDS-LVGGITNPGEVFCSVPGRLSLLSSTSK    233
mouseTFAP2E          MSLLDQSVIKKVPIPSKAG--SLSTLALSKDS-LVGGISNPSEVFCSVPGRLSLLSSTSK    233
                     :.:**:*******:*** .  ::*:*:*:*:.  :**::**.******************

zebrafishTfap2e      YKVTVGEVQRRLAPPECLNASLLGGVLRRAKSKNGGRCLRERLEKIGLNLPAGRRKAANV    285
humanTFAP2E          YKVTVGEVQRRLSPPECLNASLLGGVLRRAKSKNGGRCLRERLEKIGLNLPAGRRKAANV    293
mouseTFAP2E          YKVTVGEVQRRLSPPECLNASLLGGVLRRAKSKNGGRCLRERLEKIGLNLPAGRRKAANV    293
                     ************:***********************************************

zebrafishTfap2e      TLLTALVEGEAVHLARDFGYVCETEFPARATAEYLCRQT-EPDQLPTRRSMLLATKEICK    344
humanTFAP2E          TLLTSLVEGEAVHLARDFGYVCETEFPAKAAAEYLCRQHADPGELHSRKSMLLAAKQICK    353
mouseTFAP2E          TLLTSLVEGEAVHLARDFGYVCETEFPAKAAAEYLCRQHADPGELHSRKSMLLAAKQICK    353
                     ****:***********************:*:*******  :*.:* :*:*****:*:***

zebrafishTfap2e      EFVDLMSQDRSPLGASRPTPCLEPGVQSSLTHFSLLTHGFGTPALCAALSAFQSYLLEAL    404
humanTFAP2E          EFADLMAQDRSPLGNSRPALILEPGVQSCLTHFSLITHGFGGPAICAALTAFQNYLLESL    413
mouseTFAP2E          EFADLMAQDRSPLGNSRPALILEPGVQSCLTHFSLITHGFGGPAICAALTAFQNYLLESL    413
                     **.***:******* ***:  *******.******:***** **:****:***.****:*

zebrafishTfap2e      KLLDKGEN-------GGKNHHDKELKHRK    426
humanTFAP2E          KGLDKMFLSSVGSGHGETKASEKDAKHRK    442
mouseTFAP2E          KGLEKMFLSGAGGGHGESKASEKDTKHRK    442
                     * *:*          * .:  :*: ****
```

Supplementary Data 10: Multiple amino acid sequence alignment of zebrafish, human and mouse TFAP2E and indication of protein domains

Sequence alignment of zebrafish (Q6P0E7), human (Q6VUC0) and mouse (Q6VUP9) TFAP2E protein sequences acquired from Uniprot and aligned using Clustal Omega multiple sequence alignment (https://www.ebi.ac.uk/Tools/msa/clustalo/). Similar residues are indicated by ":", identical residues by "*". The red dotted boxes mark the human variant residues and corresponding residues in mice and zebrafish.

Note the high sequence similarity across the transactivation domain (p.48–113, chequered bar), DNA-binding domain (p.217–279, black/white striped bar) and dimerization domain (p.280-418, grey bar).

S11

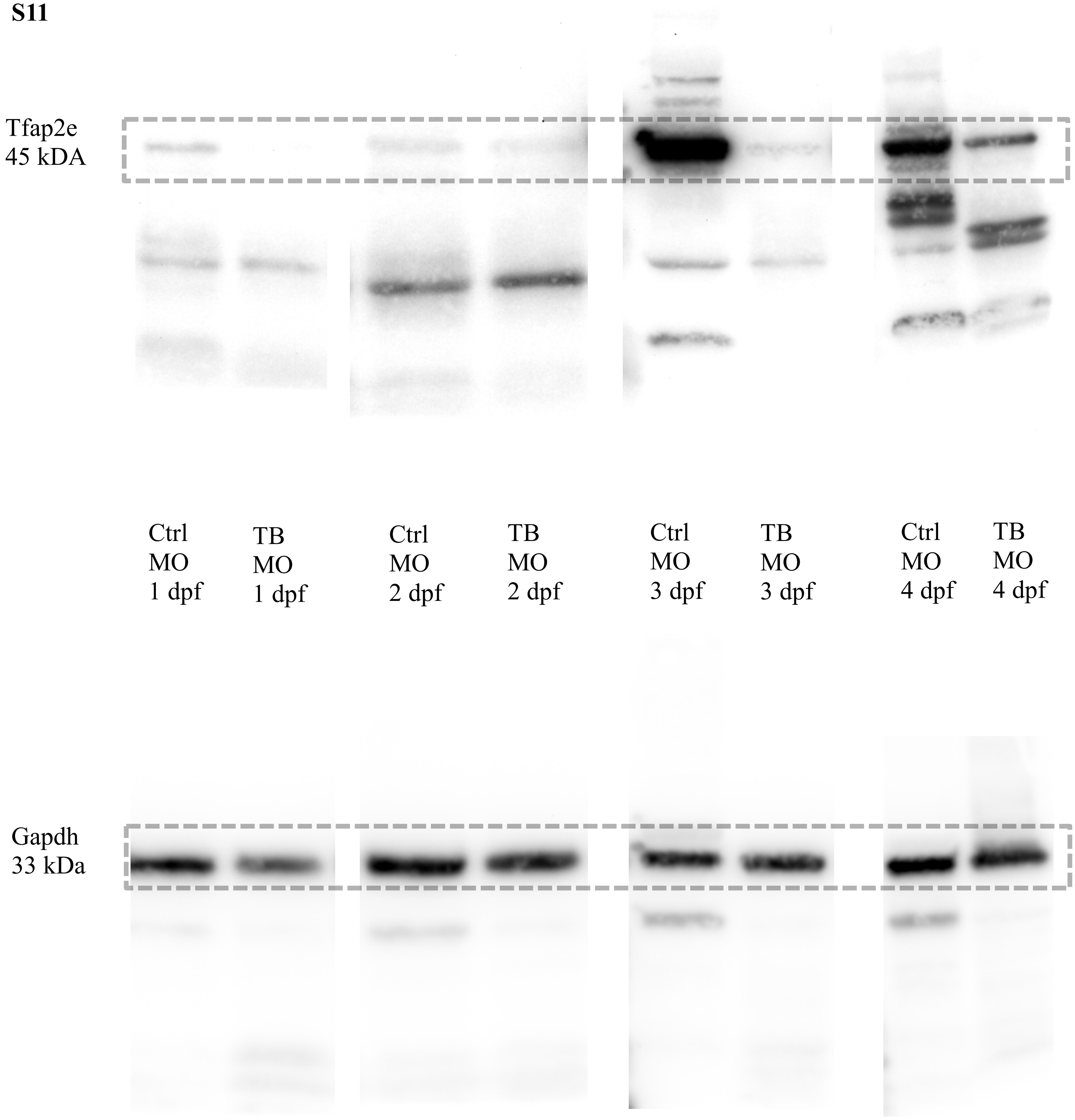

Supplementary Data 11: Images of western blot chemiluminescent membranes upon Tfap2e KD

Whole, uncropped Western Blot membranes of zebrafish larvae (zfl) protein incubated with Tfap2e antibody (upper grey box) or Gapdh antibody (lower grey box). The marked boxes are shown in Figure 2A. Zfl were treated with *tfap2e* translation blocking Morpholino (TB MO) or Control MO (Ctrl MO) from one to four days post fertilization (dpf).

S12

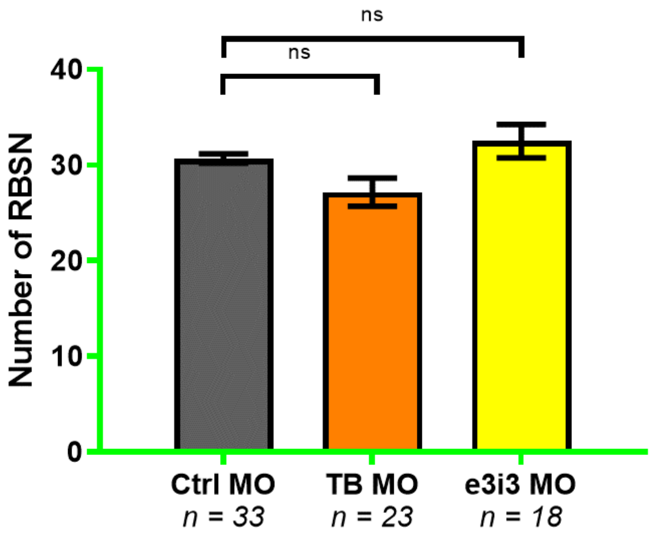

Supplementary Data 12: Quantification of Rohon-Beard sensory neurons in the spinal cord of zebrafish larvae upon Tfap2e KD

Column graph of counted Rohon-Beard sensory neurons (RBSN) after *in vivo* 2-photon microscopy of the spinal cord of *Tg(-3.1ngn1:GFP)* zebrafish larvae (zfl) upon Tfap2e knockdown (KD) at two days post fertilization (dpf). We acquired z-stack images of the spinal cord with a step size of 2 µm. Representative images are provided in FIGURE 3F. Note the non-significant affection in e3i3 MO treated zfl and TB MO treated zfl compared to controls (Ctrl MO). This stands in contrast to the more severe affection of the dorsal root ganglia (DRG, FIGURE 3G).

Ns not significant, * $p < 0.05$ [one-way ANOVA with Tukey's multiple comparison]. Number of independent experiments $N = 3$, number of investigated zfl n as indicated.

S13

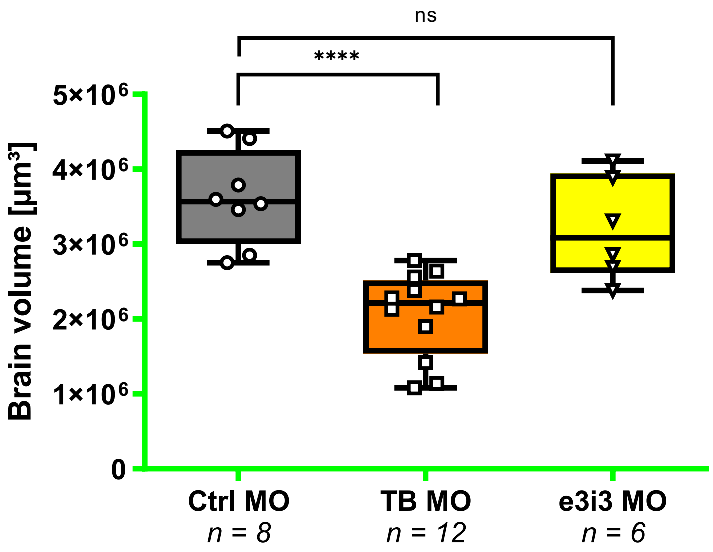

Supplementary Data 13: Statistical analysis of the microencephaly of zebrafish larvae upon Tfap2e KD

Box plot graph of the microencephaly phenotype following Tfap2e KD in zfl, subsequent *in vivo* 2-photon z–stack imaging and modulation with IMARIS software. Representative 3D-modelling images and videos of the brain of *Tg(-3.1ngn1:GFP)* zebrafish larvae (zfl) upon Tfap2e knockdown (KD) and controls at two days post fertilization (dpf) are provided in FIGURE 4B and in Supplementary Video 1-3. TB MO KD in zfl but not e3i3 MO KD led to a reduced brain volume consistent with a microencephaly.

**** $p < 0.0001$ [one-way ANOVA with Tukey's multiple comparison]. Number of independent experiments $N = 3$ for all graphs, number of investigated zfl n as indicated.

S14

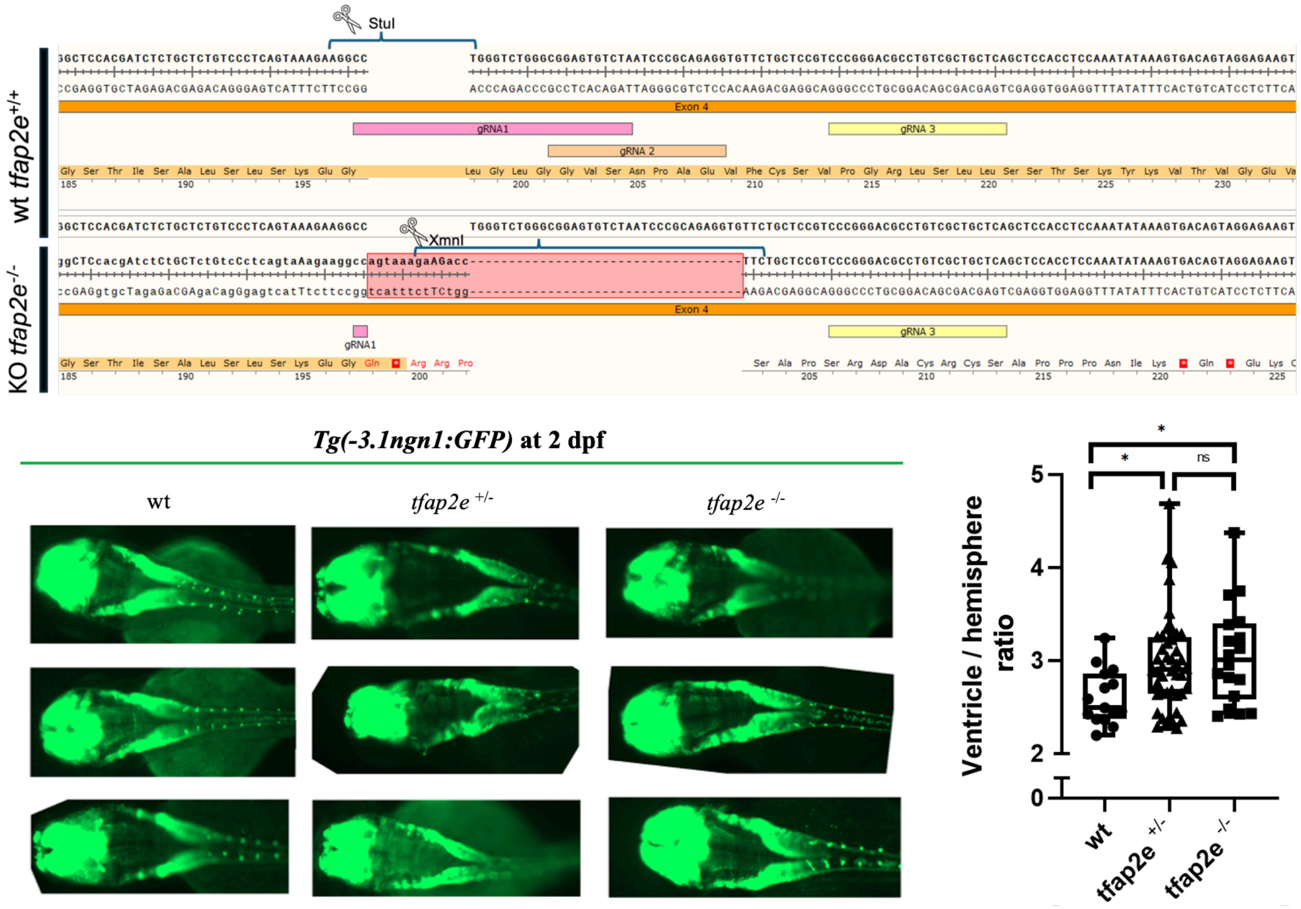

Supplementary Data 14: CRISPR/Cas9 mediated knockout of *tfap2e* in zebrafish larvae leads to hydrocephalus

Upper panel: Screenshot from snapgene viewer. Top row: Wildtype *tfap2e* sequence with corresponding amino acid sequence. Positions of the binding sites of guideRNAs 1-3 (gRNA) as indicated. The binding site of gRNA4 is downstream of the depicted section. Bottom row: DNA and amino acid sequence of *tfap2e*$^{-/-}$ KO^{CRISPR} zfl. The injection of the gRNA mix leads to an insertion of 13bp and deletion of 35bp in exon 4, resulting in a nonsense mutation. This indel leads to disruption of the StuI binding site (5'AGGCCT3') and introduces a new XmnI binding site (5'GAANNNNTTC3'). Double digestion with XmnI and StuI was performed after PCR amplification of exon 4 for genotyping.

Lower panel: *Left*: Representative images of tfap2e$^{+/-}$, tfap2e$^{-/-}$ zfl and wt littermate controls at two dpf. Zfl were anaesthetized and mounted dorsally. Hydrocephalus was observed in both, *tfap2e*$^{+/-}$ and tfap2e$^{-/-}$ zfl. Note that only a subset of zfl present with hydrocephalus, pointing towards a not fully penetrant phenotype. *Right*: Representative box plot graph of the hydrocephalus seen in *tfap2e*$^{+/-}$ (n=44, N=3) and *tfap2e*$^{-/-}$ (n=18, N=3) KO^{CRISPR} zfl, compared to littermate wt$^{+/+}$ controls (n=15, N=3). To account for variation in head size, the ventricle diameter was put in relation to the diameter of the brain hemisphere, analogous to FIGURE 4D. Ns not significant. * $p < 0,05$ [one-way ANOVA].

gRNA1: CCTGGGTCTGGGCGGAGTGTCTA. gRNA2: CGGAGTGTCTAATCCCGCAGAGG. gRNA 3: CCCGGGACGCCTGTCGCTGCTCA. gRNA4: CCTCAACGCCTCGCTGCTTGGAG.

Design of sgRNAs targeting *tfap2e*, founder identification and delivery of F1 animals was commercially performed by Nanjing Sanjay Medical Technology Co (Nanjing, China)

Supplementary Videos 1-3:

Representative 3D – modelling video of the brain of *Tg(-3.1ngn1:GFP)* zebrafish larvae (zfl) upon TB MO (Supplementary Video 2), e3i3 MO (Supplementary Video 3) *tfap2e* knockdown (KD) or Ctrl MO (Supplementary Video 1) at two days post fertilization (dpf). The representative images were obtained by *in vivo* 2-photon z–stack imaging and subsequent modulation with IMARIS software. 360° rotation around the y-axis is followed by 360° rotation around the x-axis. Transverse (left) and coronary view (right). Again, note the microcephaly of TB MO treated zfl (Supplementary Data 13) and the enlarged hemisphere cleft of e3i3 MO treated zfl (FIGURE 4C).
