# Supplementary material for: TFAP2E is implicated in central nervous system, orofacial and maxillofacial anomalies
Source: J Med Genet. 2024 Dec 23;62(2):e109799. doi: 10.1136/jmg-2023-109799 (PMC11777392; doi:10.1136/jmg-2023-109799)
Supplement: online supplemental file 2 [file jmg-62-2-s002.pdf]

Family A

TFAP2E WT versus p.Ala8Asp

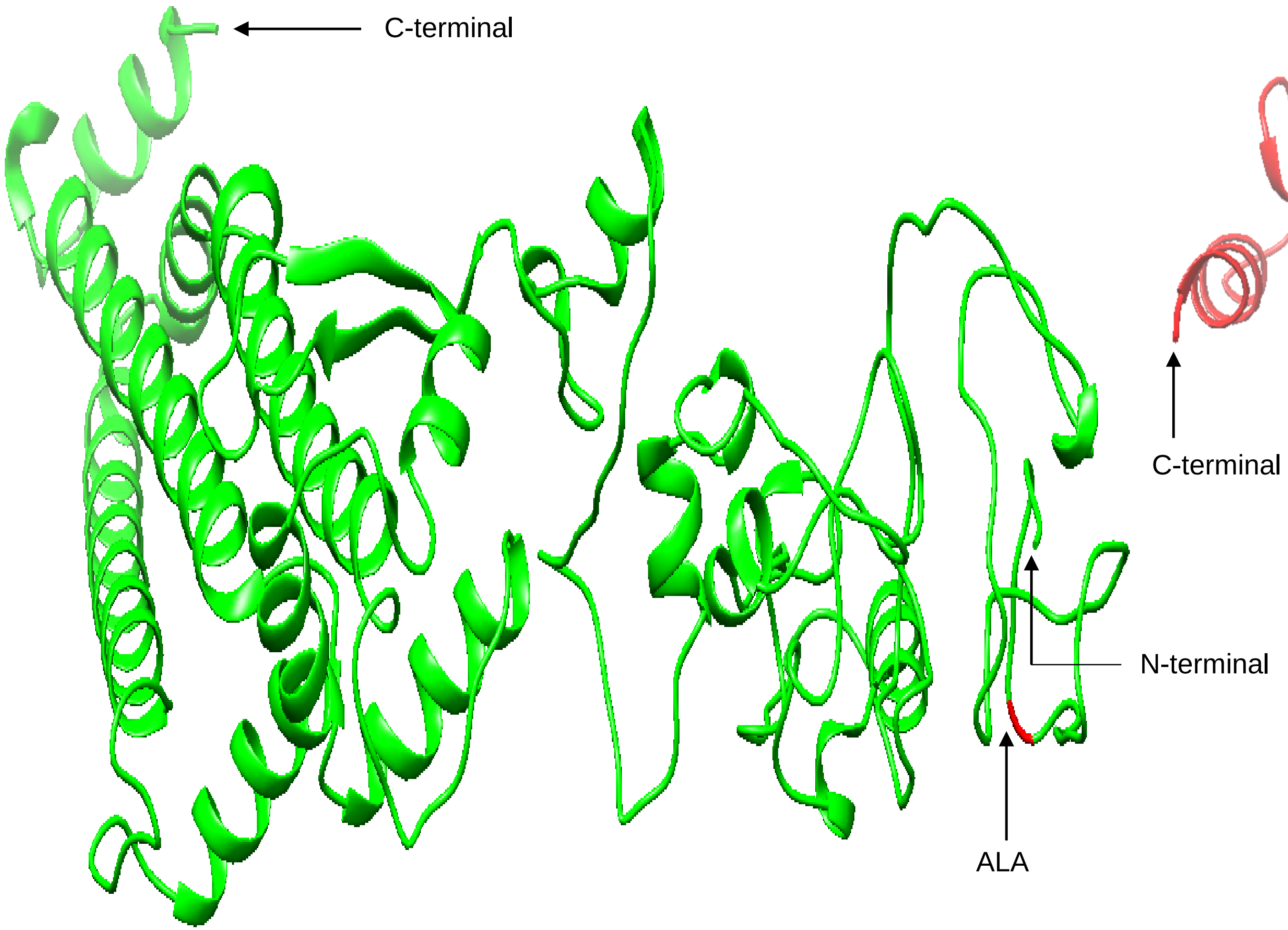

WILD_TFAP2E

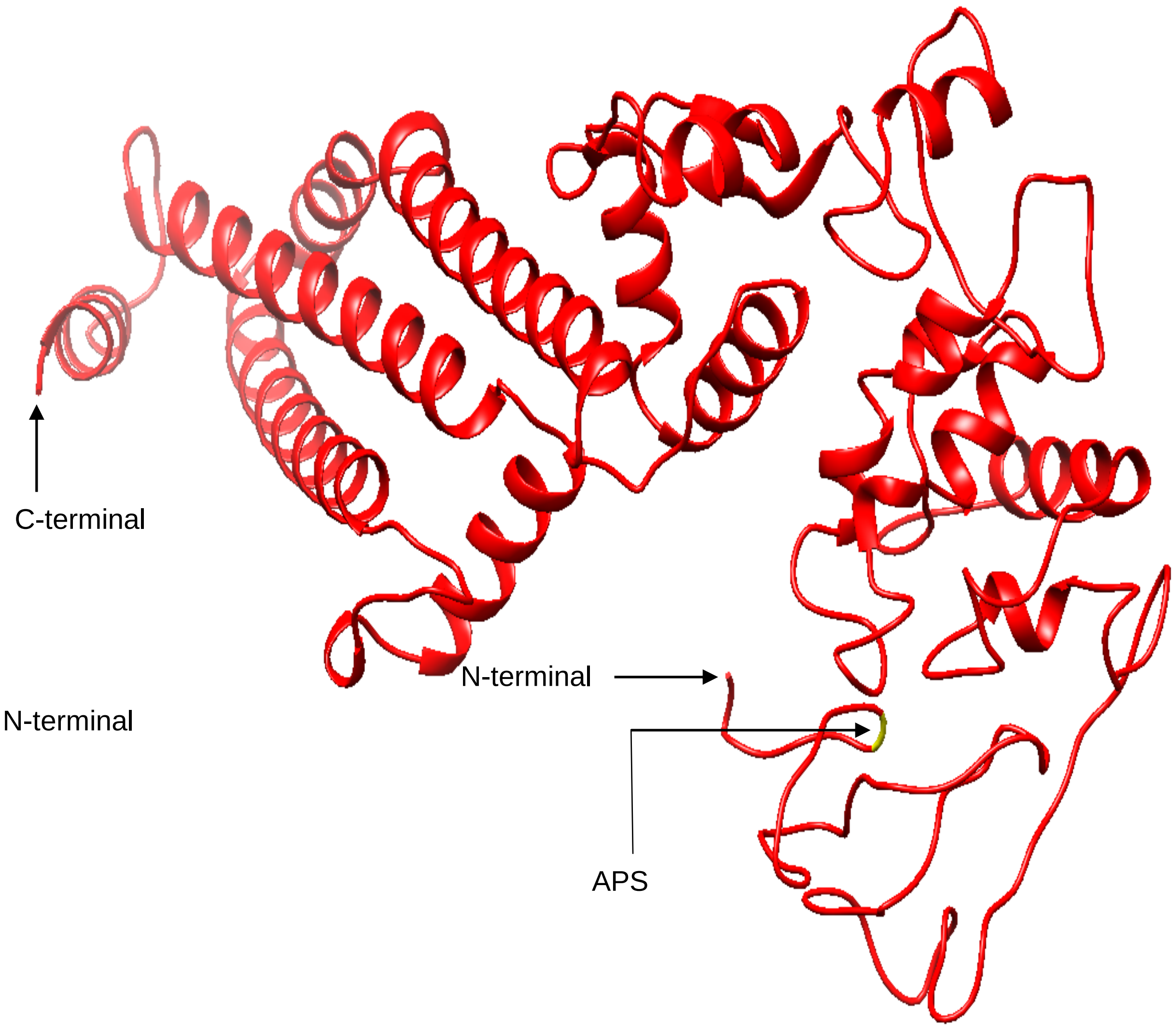

MUTANT_1_TFAP2E
ALA8ASP

Family A

TFAP2E WT versus p.Ala8Asp

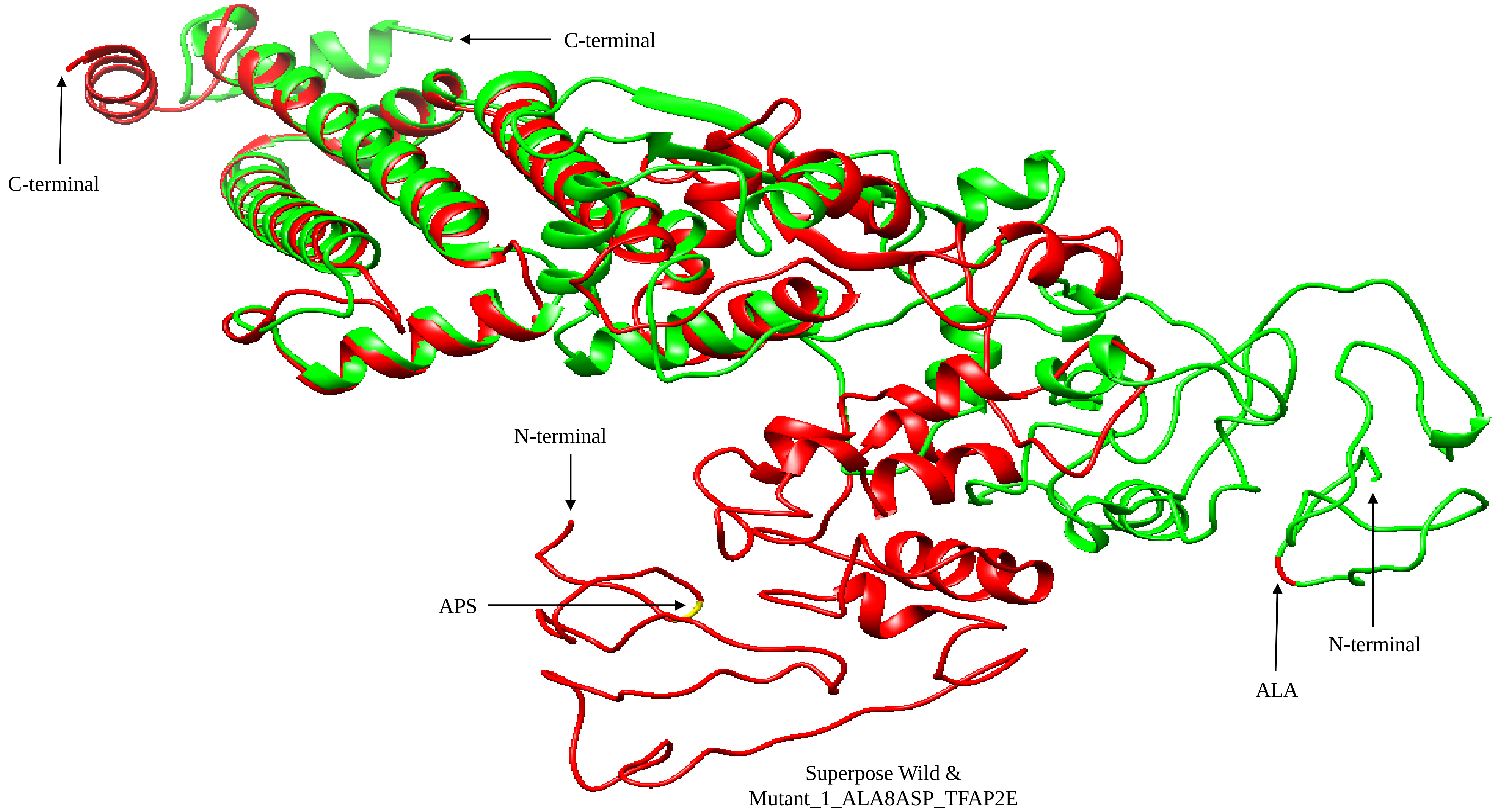

Family B

TFAP2E WT versus p.Pro113 Thr

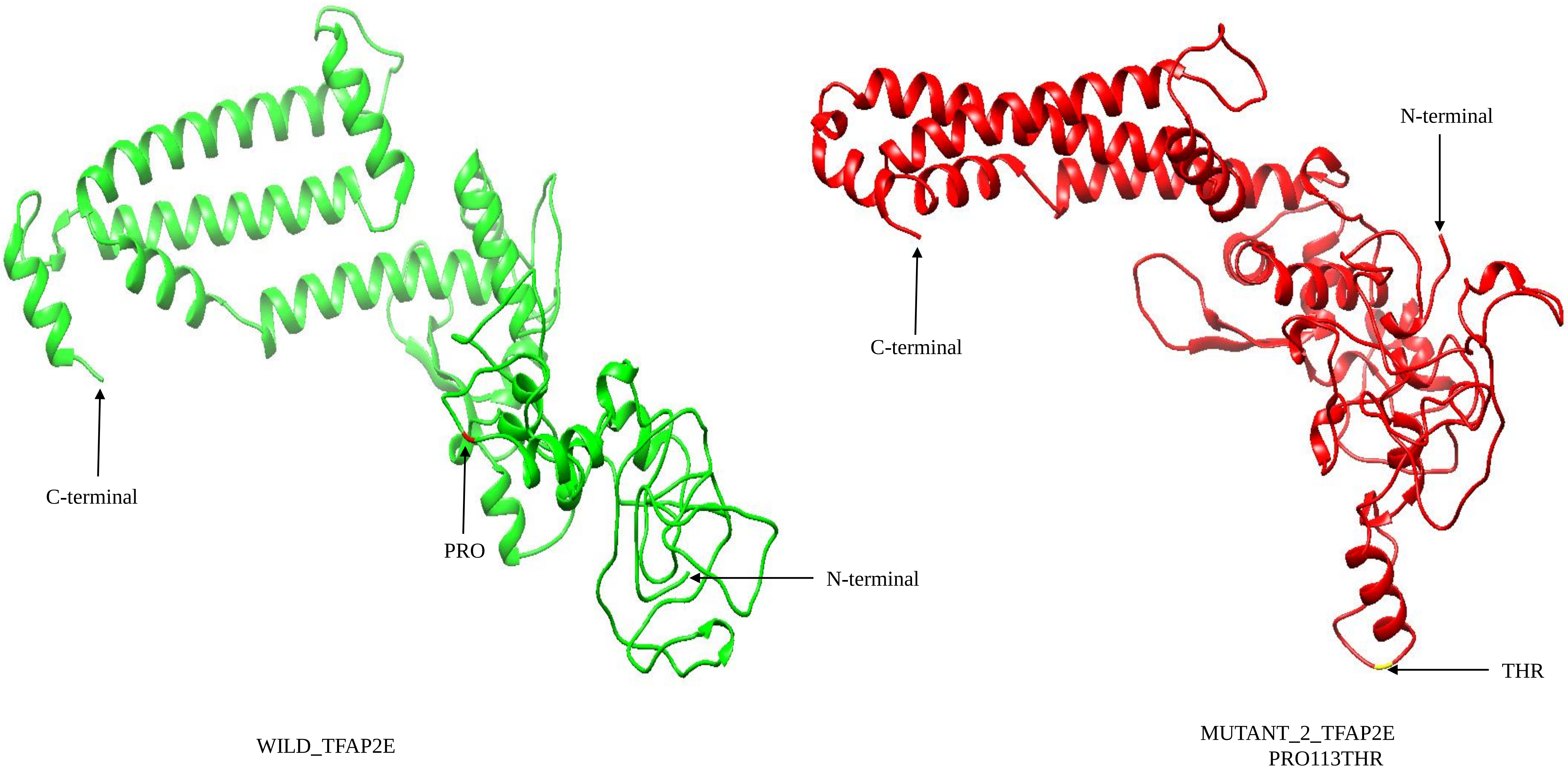

Family B

TFAP2E WT versus p.Pro113 Thr

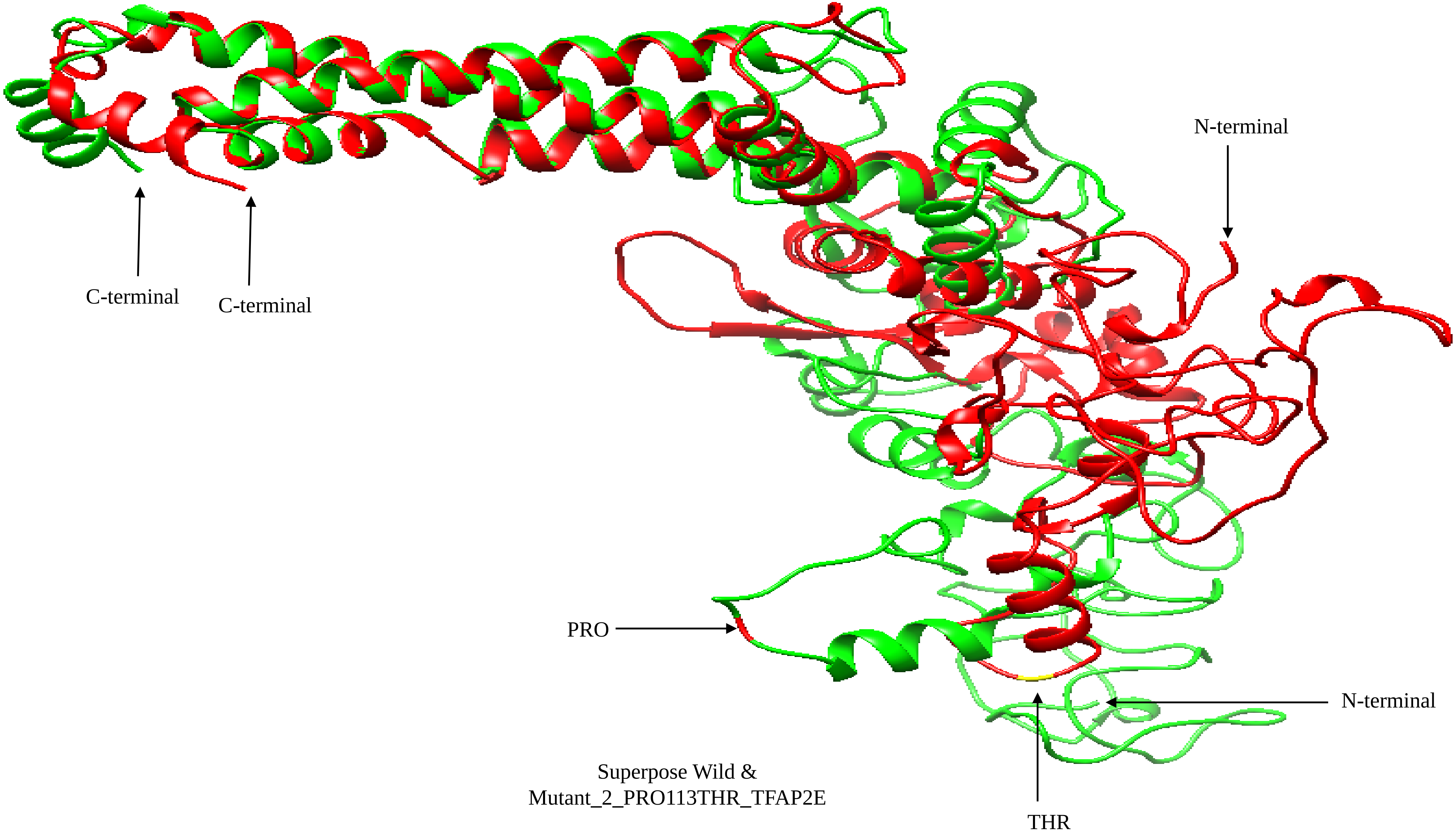

Superpose Wild &
Mutant_2_PRO113THR_TFAP2E

Family B

TFAP2E WT versus p.Pro113 Thr

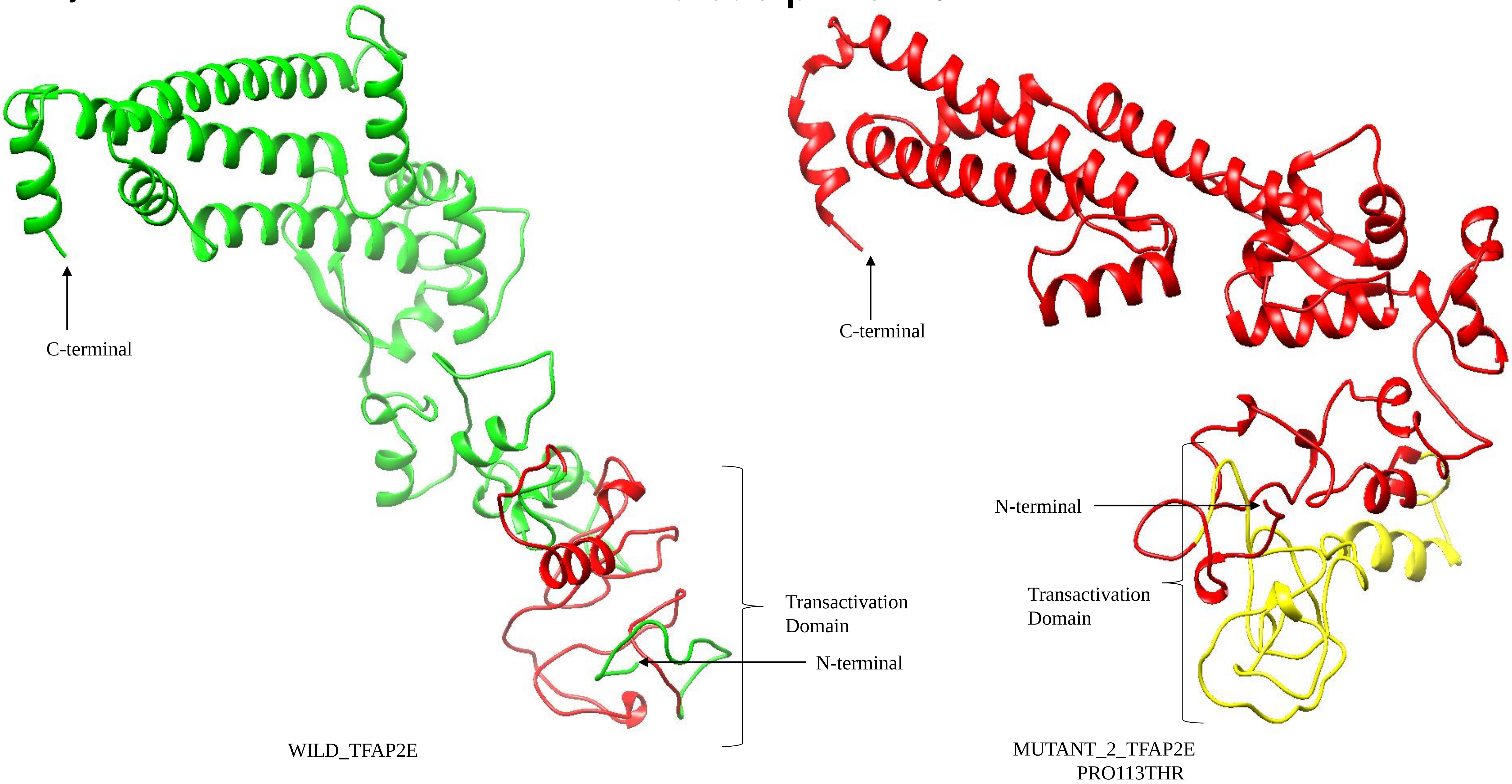

Family C

TFAP2E WT versus p.Arg129His

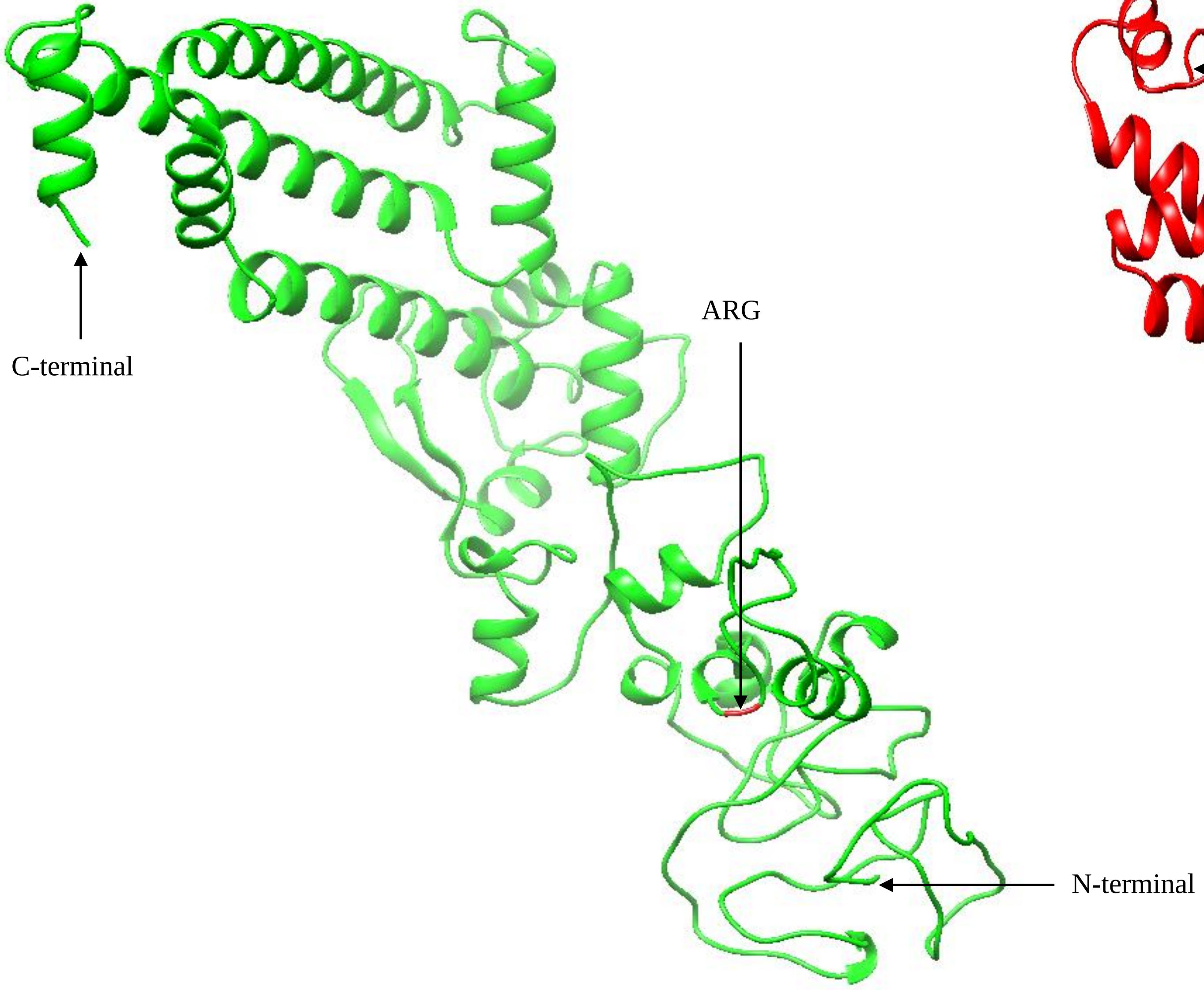

WILD_TFAP2E

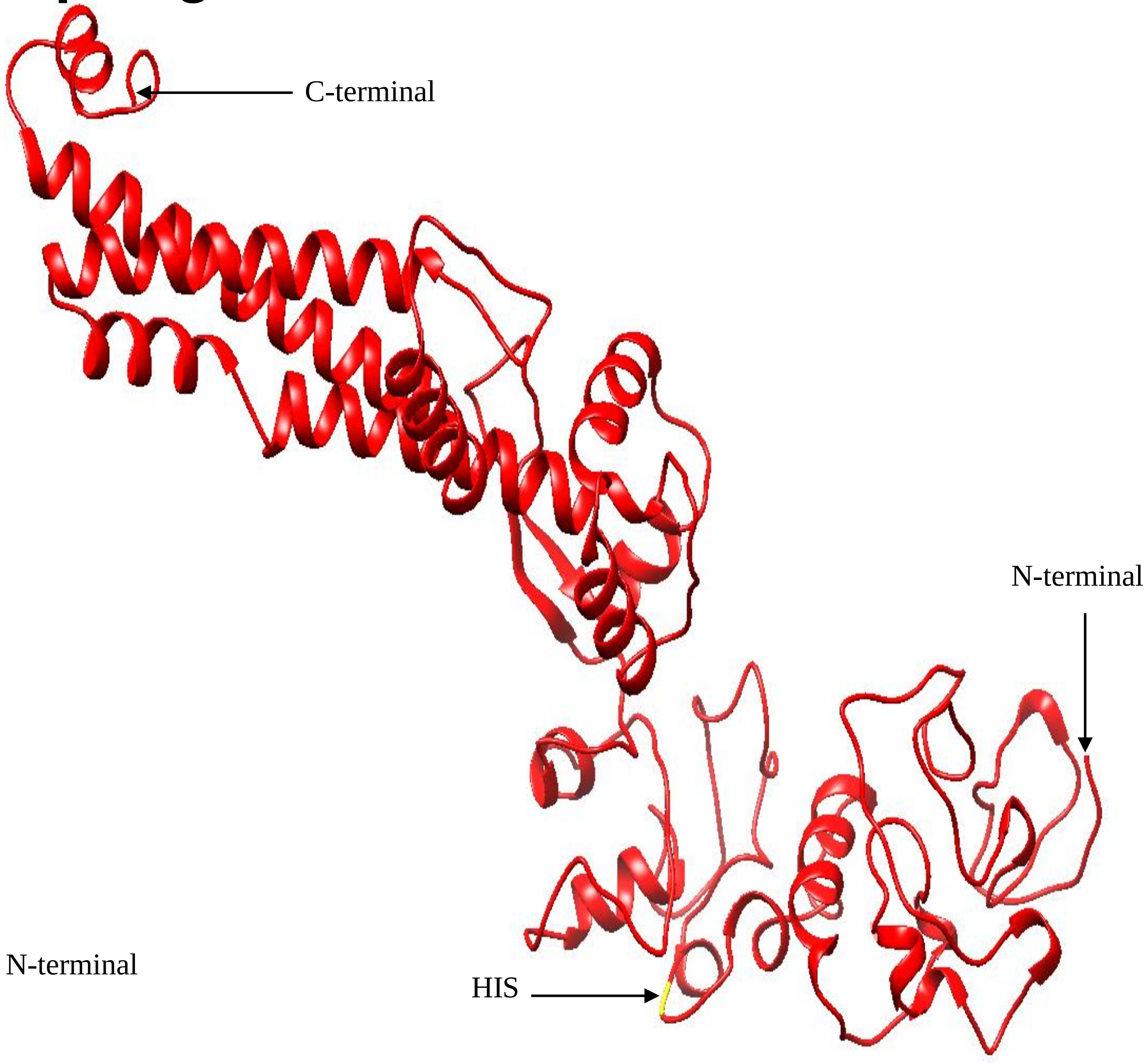

MUTANT_3_TFAP2E
ARG129HIS

Family C

TFAP2E WT versus p.Arg129His

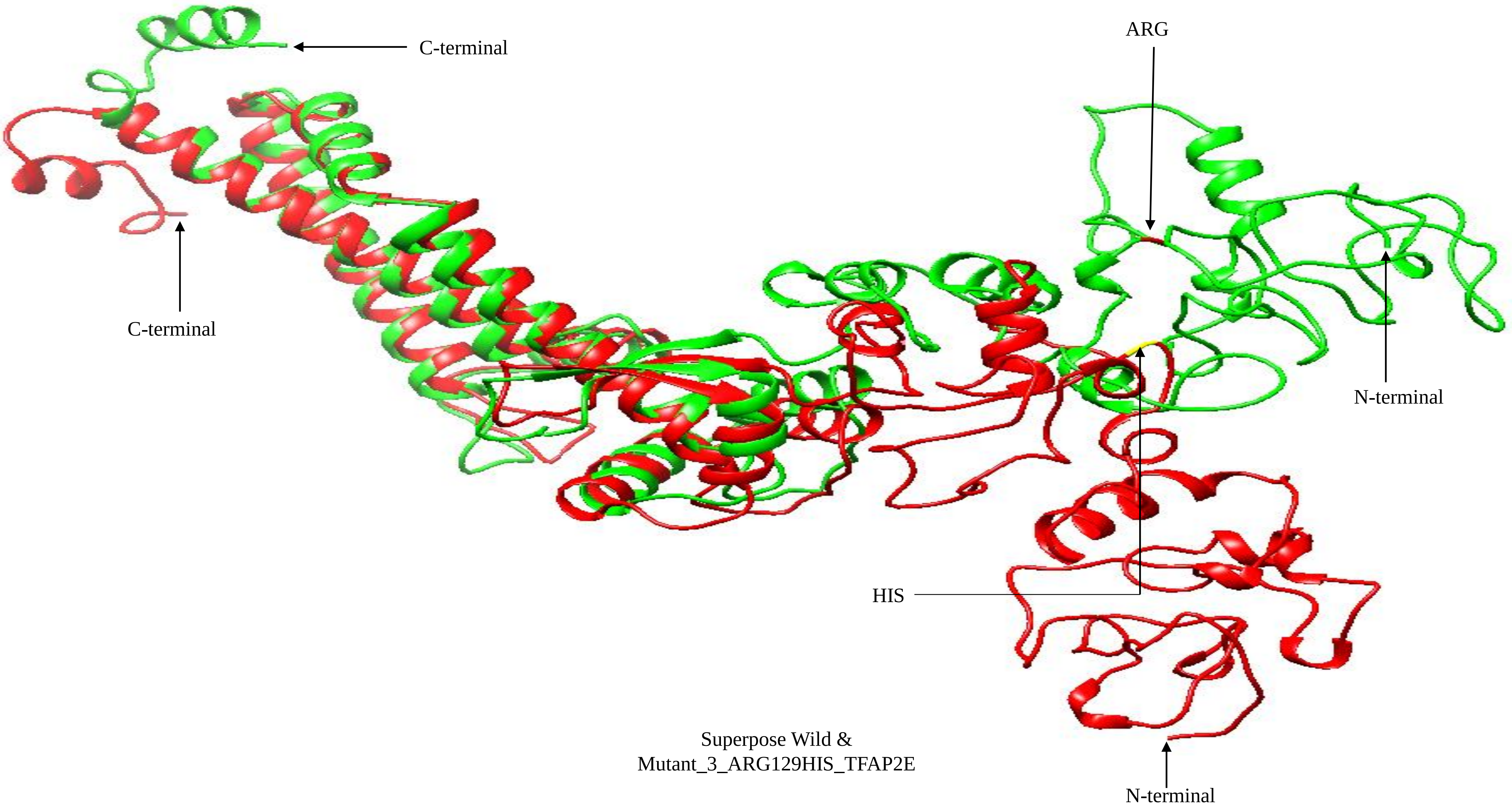

Superpose Wild &
Mutant_3_ARG129HIS_TFAP2E

Family D

TFAP2E WT versus p.Leu228Phe

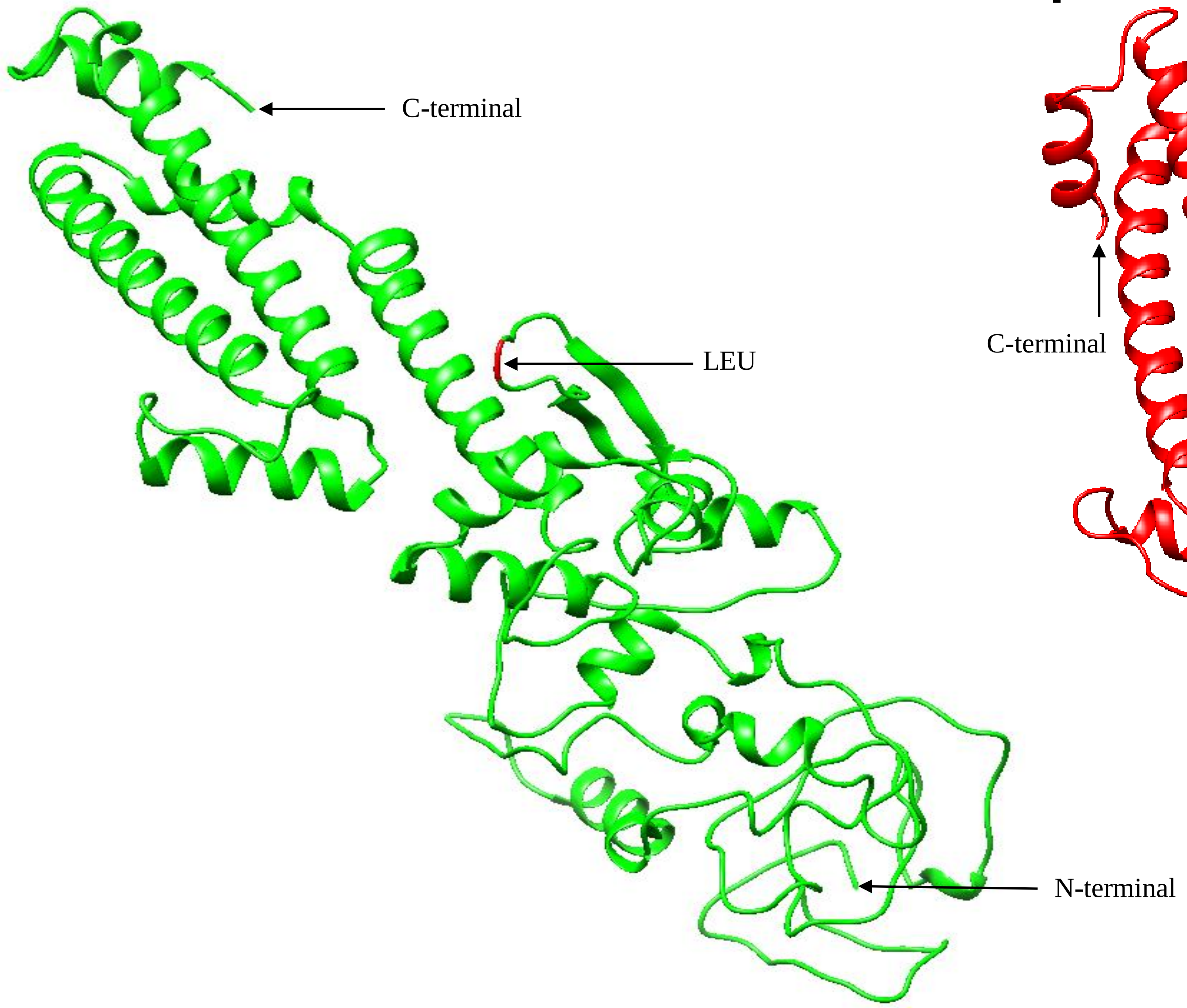

WILD_TFAP2E

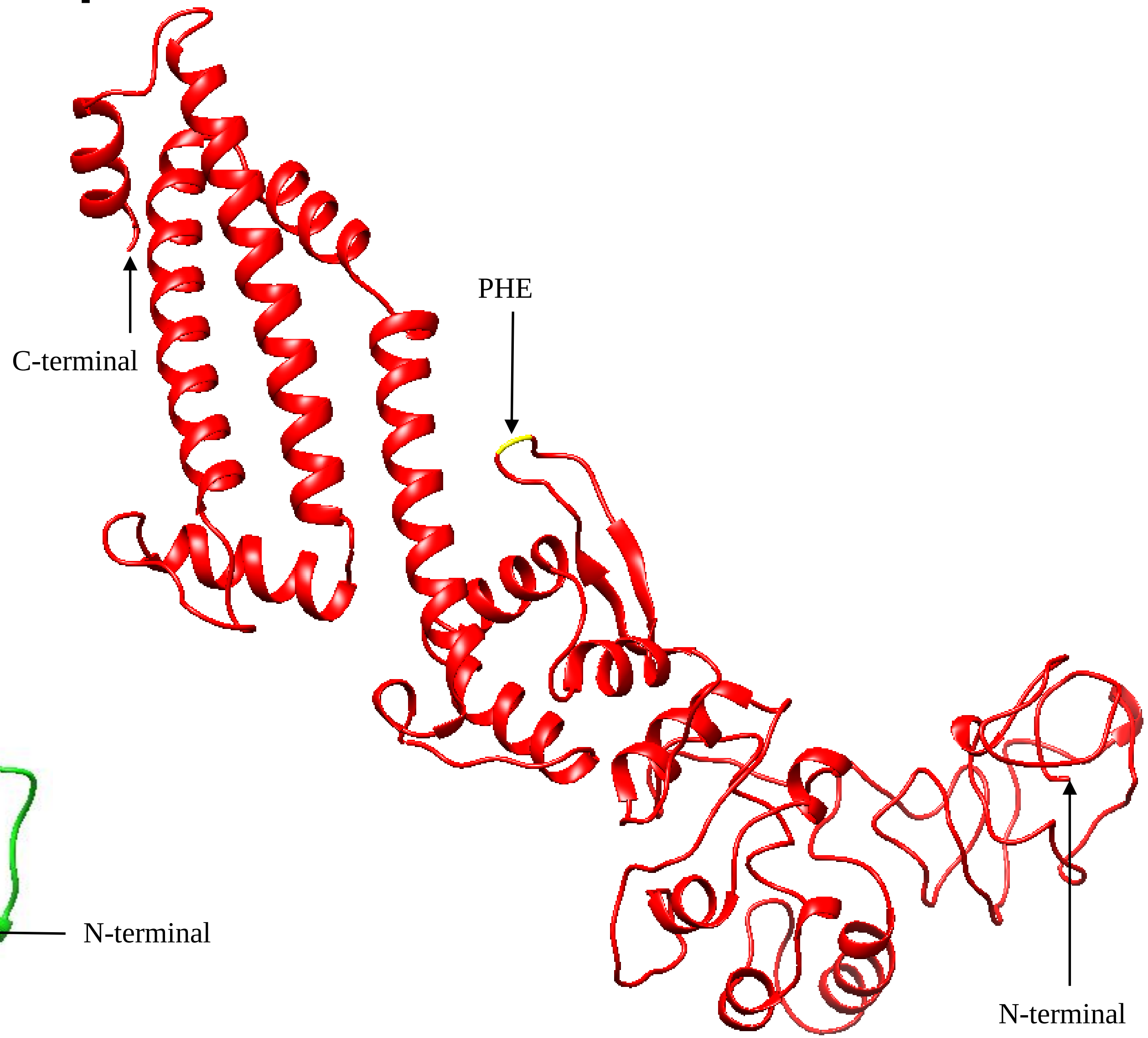

MUTANT_4_TFAP2E
LEU228PHE

Family D

TFAP2E WT versus p.Leu228Phe

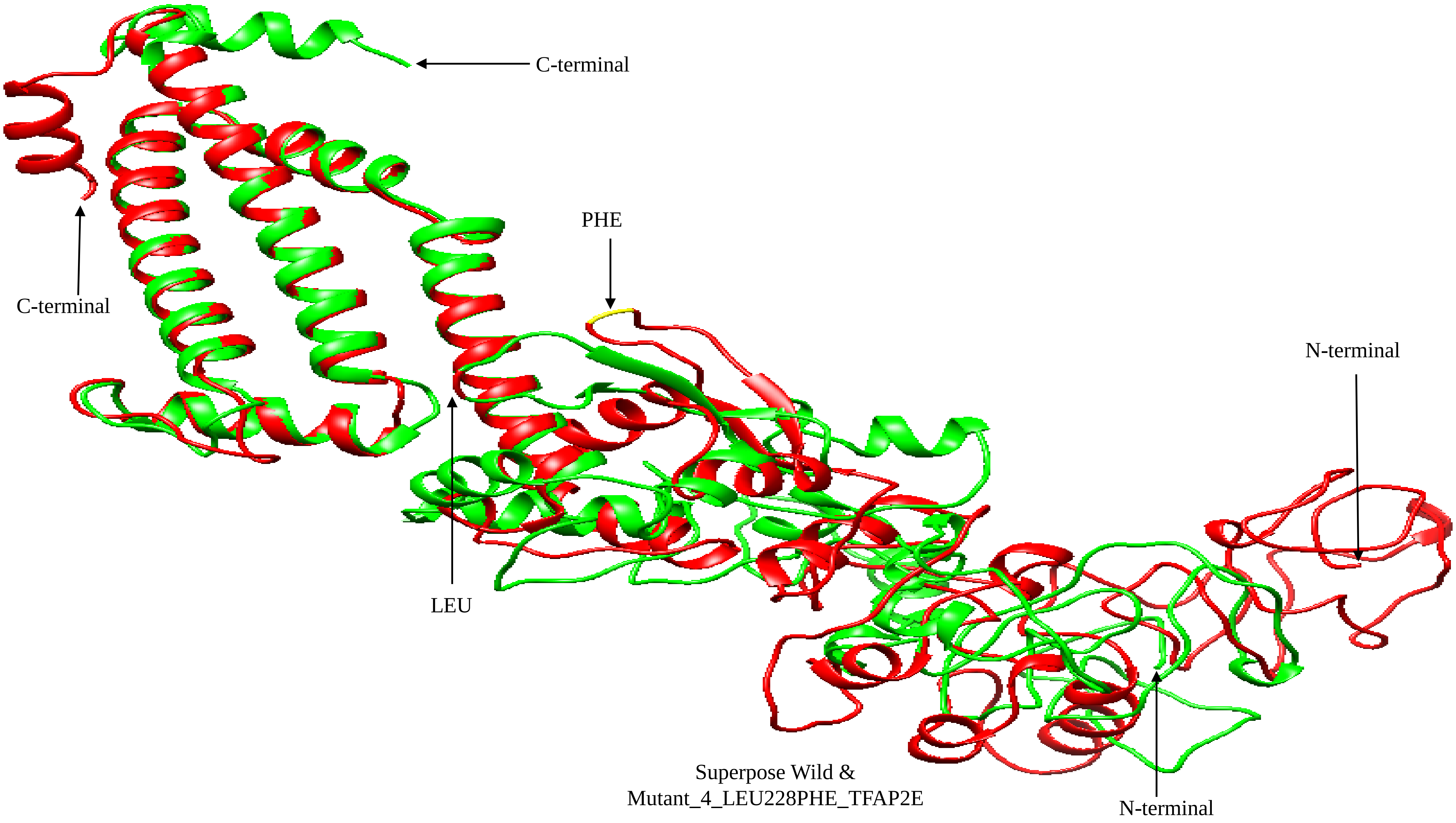

Family E

TFAP2E WT versus p.Gly312Ser

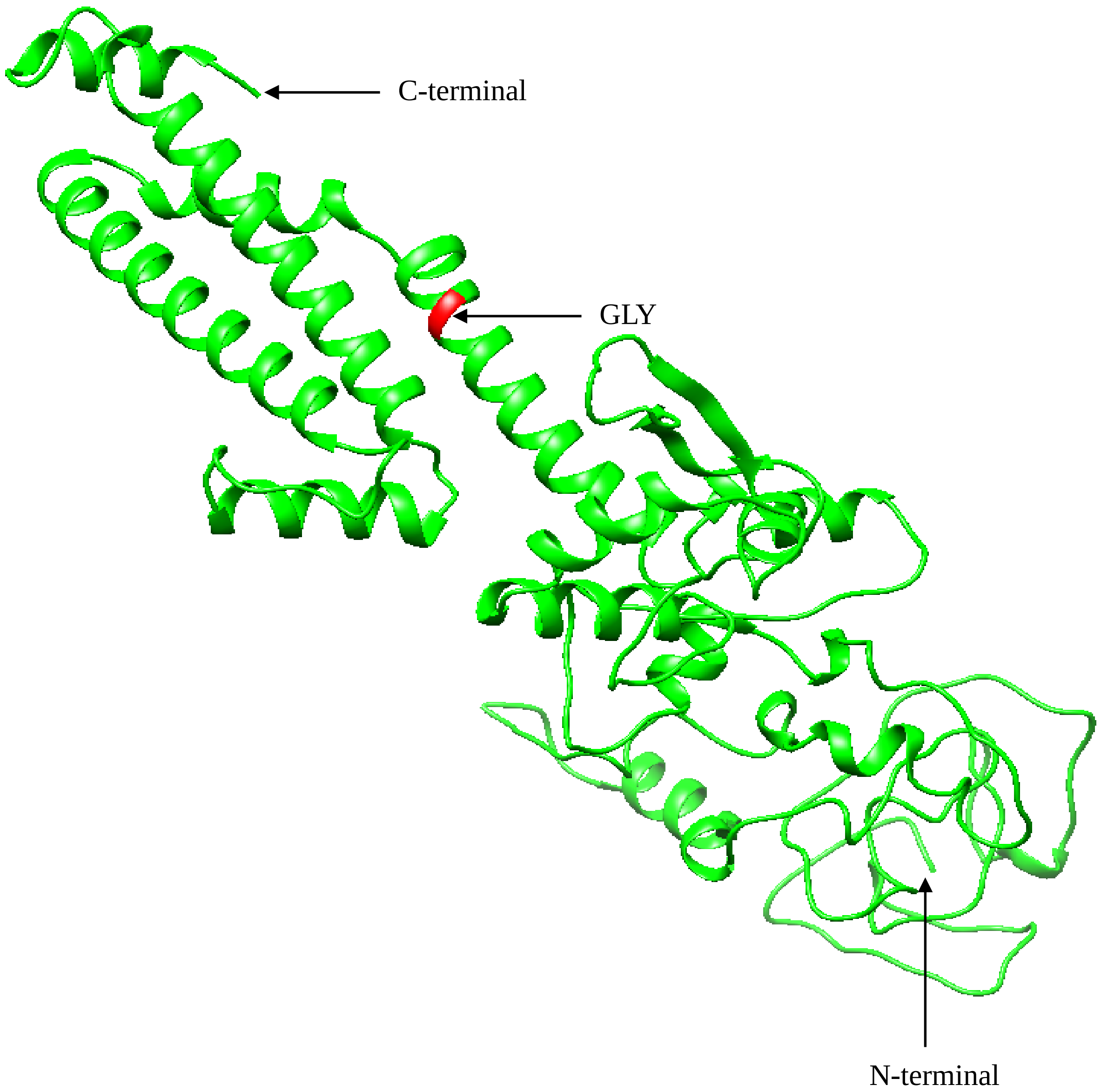

WILD_TFAP2E

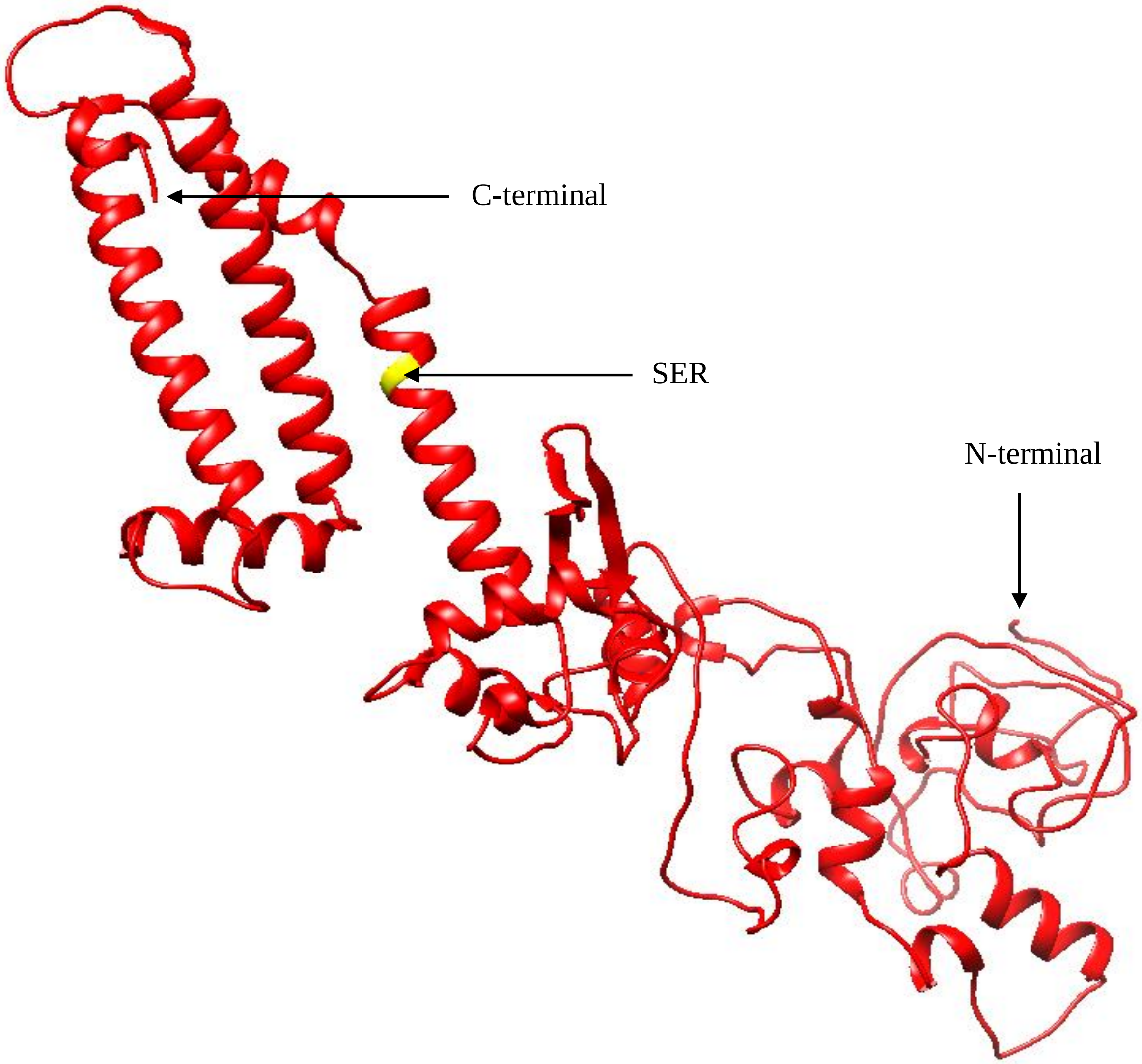

MUTANT_5_TFAP2E
GLY312SER

Family E

TFAP2E WT versus p.Gly312Ser

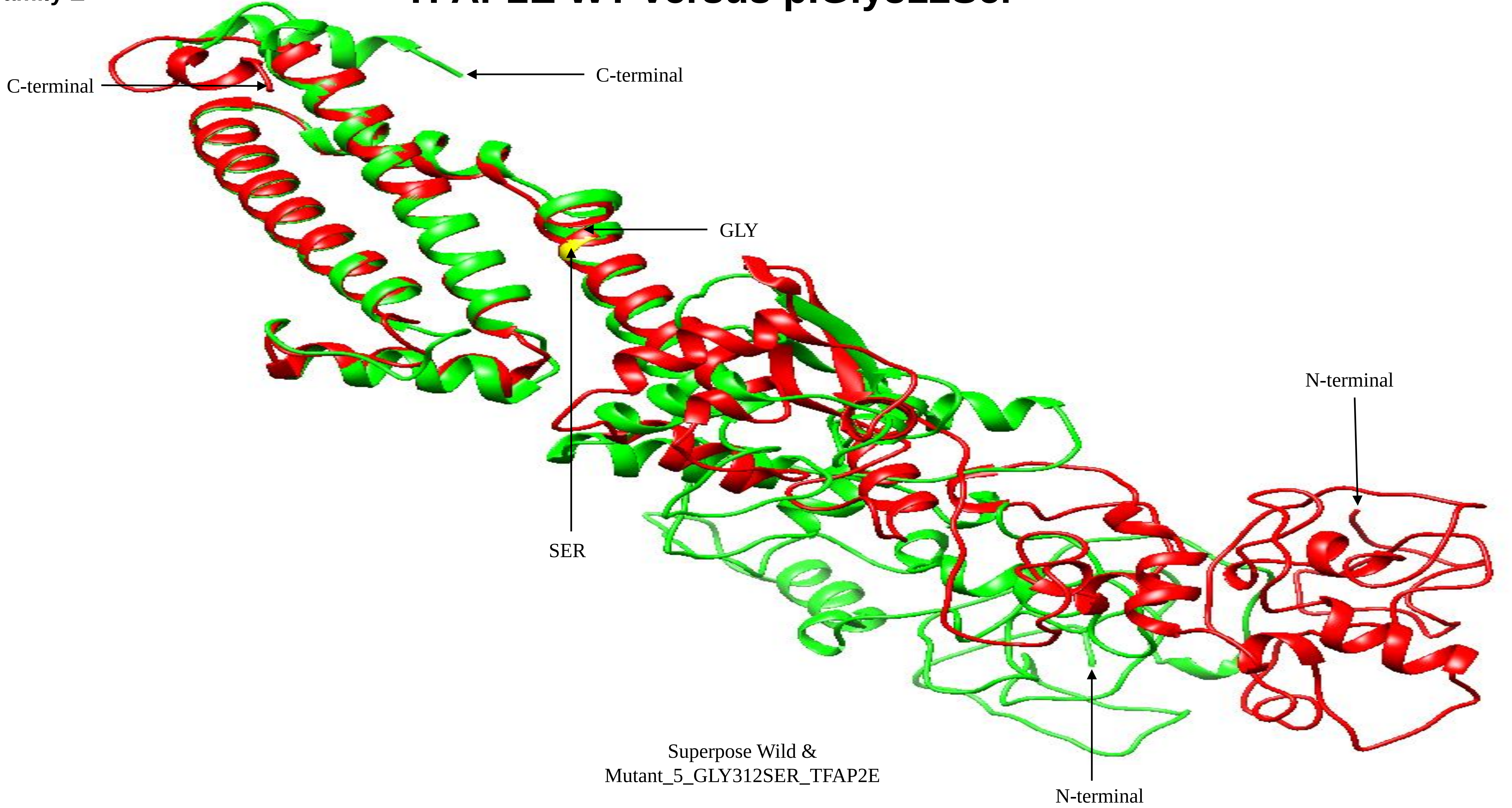

Superpose Wild &
Mutant_5_GLY312SER_TFAP2E
