# Supplementary material for: TFAP2E is implicated in central nervous system, orofacial and maxillofacial anomalies
Source: J Med Genet. 2024 Dec 23;62(2):e109799. doi: 10.1136/jmg-2023-109799 (PMC11777392; doi:10.1136/jmg-2023-109799)
Supplement: online supplemental table 1 [file jmg-62-2-s003.pdf]

Family ID	Variant	Structure Amino acid position	Amino acid-structure wildtype	Amino acid-structure variant	Metadome protein tolerance landscape
A	p.Ala8Asp	8	A – Loop	D - Loop	highly tolerant
A	p.Ala8Asp	47-49	AAE - Helix	AAE - Loop	intolerant - highly intolerant
B	p.Pro113Thr	50	F - Loop	F - Helix	highly intolerant
B	p.Pro113Thr	96-99	PQAA - Helix	PQAA - Loop	intolerant - highly intolerant
B	p.Pro113Thr	113	P – Loop	T - Loop	highly tolerant
B	p.Pro113Thr	123-126	ALGL - Loop	ALGL - Helix	slightly tolerant - neutral
B	p.Pro113Thr	128-130	PRR - Loop	PRR - Helix	neutral - slightly intolerant
C	p.Arg129His	120-124	PARAL - Loop	PARAL - Helix	slightly tolerant - slightly intolerant
C	p.Arg129His	129	R – Loop	H - loop	slightly intolerant
C	p.Arg129His	134	T - Loop	T - Helix	slightly tolerant
D	p.Leu228Phe	222	P - Sheet	P - Loop	neutral
D	p.Leu228Phe	228	L – Loop	F - Loop	tolerant
D	p.Leu228Phe	233	K - Sheet	K - Loop	slightly tolerant
D	p.Leu228Phe	245	L - Loop	L - Helix	intolerant
E	p.Gly312Ser	294	T - Helix	T - Loop	highly tolerant
E	p.Gly312Ser	312	G – Helix	S - Helix	slightly intolerant
E	p.Gly312Ser	332	H - Helix	H - Loop	slightly intolerant
